# In Silico Functional Prediction and Expression Analysis of C2H2 Zinc-Finger Family Transcription Factor Revealed Regulatory Role of *ZmZFP126* in Maize Growth

**DOI:** 10.3389/fgene.2021.770427

**Published:** 2021-11-05

**Authors:** Jia Li, Litian Zhang, Yibing Yuan, Qi Wang, Rania G. Elbaiomy, Wanhai Zhou, Hui Wu, Salma A. Soaud, Manzar Abbas, Bo Chen, Deming Zhao, Ahmed H. El-Sappah

**Affiliations:** ^1^ Faculty of Agriculture, Forestry and Food Engineering, Yibin University, Yibin, China; ^2^ State Key Laboratory of Plateau Ecology and Agriculture, Academy of Animal Science and Veterinary Medicine of Qinghai University, Xining, China; ^3^ Maize Research Institute, Sichuan Agricultural University, Chengdu, China; ^4^ Key Laboratory of Biology and Genetic Improvement of Maize in Southwest Region, Ministry of Agriculture, Chengdu, China; ^5^ State Key Laboratory of Crop Gene Exploration and Utilization in Southwest China, Chengdu, China; ^6^ Faculty of Pharmacy, Ahram Canadian University, Cairo, Egypt; ^7^ Genetics Department, Faculty of Agriculture, Zagazig University, Zagazig, Egypt; ^8^ Yibin Academy of Agricultural Sciences, Yibin, China

**Keywords:** maize, C2H2 zinc finger gene family, C2H2-ZF domain, phylogenetic analysis, association analysis

## Abstract

The C2H2-zinc finger proteins (ZFP) comprise a large family of transcription factors with various functions in biological processes. In maize, the function regulation of C2H2- zine finger (ZF) genes are poorly understood. We conducted an evolution analysis and functional prediction of the maize C2H2-ZF gene family. Furthermore, the *ZmZFP126* gene has been cloned and sequenced for further favorable allelic variation discovery. The phylogenetic analysis of the C2H2-ZF domain indicated that the position and sequence of the C2H2-ZF domain of the poly-zinc finger gene are relatively conserved during evolution, and the C2H2-ZF domain with the same position is highly conserved. The expression analysis of the C2H2-ZF gene family in 11 tissues at different growth stages of B73 inbred lines showed that genes with multiple transcripts were endowed with more functions. The expression analysis of the C2H2-ZF gene in P1 and P2 inbred lines under drought conditions showed that the C2H2-ZF genes were mainly subjected to negative regulation under drought stress. Functional prediction indicated that the maize C2H2-ZF gene is mainly involved in reproduction and development, especially concerning the formation of important agronomic traits in maize yield. Furthermore, sequencing and correlation analysis of the *ZmZFP126* gene indicated that this gene was significantly associated with the SDW-NAP and TDW-NAP. The analysis of the relationship between maize C2H2-ZF genes and C2H2-ZF genes with known functions indicated that the functions of some C2H2-ZF genes are relatively conservative, and the functions of homologous genes in different species are similar.

## Introduction

Transcription factors play an important role in plant and animal response to various internal or external factors in order to adapt to the environment. In 1985, three research teams independently described the DNA/RNA-binding transcription factor TFIIIA in *Xenopus laevis*, and it contains nine conserved repeats of cysteine (C), histidine (H), and hydrophobic amino acid residues (Ginsberg et al., 1984; Brown et al., 1985; Miller et al., 1985). The arrangement of conserved amino acids in TFIIIA is $-XC-X_2,4,5_-C-X_3_-$-X_5_-$-X_2_-H-X_3,4_-H, where X and $ represent any amino acid and a hydrophobic residue, respectively. Based on this observation, along with the findings of earlier biochemical studies, Klug et al. proposed the term “zinc finger” to describe that this 30-amino acid sequence motif forms an independent, DNA-binding mini-domain folded around a central zinc ion with a tetrahedral arrangement of cysteine and histidine metal ligands (Miller et al., 1985; Klug and Schwabe, 1995), The three-dimensional structure of which comprised two parallel β-sheets and an α-helix that binds to the stable structure of a zinc ion (Takatsuji, 1999). Transcription factors that contain a zinc finger domain and allow the protein to interact with DNA are termed zinc finger proteins (ZFP), which can be classified according to the number and order of cysteine and histidine residues that bind to zinc ions. The C2H2-ZFP is one of the most abundant transcription factors among the different ZFP types in eukaryotes. Bioinformatics analyses have revealed that approximately 3% of mammalian genes (Bateman et al., 2004), 2.3% of dipteran genes (Chung et al., 2002), 0.8% of *Saccharomyces cerevisiae* genes (Böhm et al., 1997), 0.4% of rice genes (Agarwal et al., 2007), 0.7% of *Arabidopsis thaliana* genes (Englbrecht et al., 2004), and 0.49% of maize (*Zea mays*) genes (Wei et al., 2016) encode the C2H2-ZFP.

Plant C2H2-ZFPs generally contain 1–5 C2H2-ZF domains. Two main characteristics distinguish the majority of plant C2H2-ZFPs from those of other eukaryotes. The first characteristic is that several plant C2H2-ZFPs contain a conserved QALGGH motif between the second C and first H of the ZF domain, whereas this QALGGH motif is absent in the C2H2-ZFPs of yeasts and animals. The second characteristic is that the space between the ZF domains is longer than that of C2H2-ZFPs of eukaryotic organisms containing multiple ZFs (Takatsuji, 1999; Ciftci-Yilmaz and Mittler, 2008). These two characteristics play an important role in the affinity of the C2H2-ZFP for binding to its target DNA (Takatsuji and Matsumoto, 1996; Kubo et al., 1998; Ciftci-Yilmaz and Mittler, 2008). According to the number, type, and arrangement pattern of ZFs, C2H2-ZFPs can be divided into three types, namely, Classes A–C (Böhm et al., 1997; Englbrecht et al., 2004). Through whole-genome comparison of *A. thaliana* using bioinformatics, it has been found that both Classes A and B contain tandem ZFs. Class C ZFPs consistently exhibit single or dispersed ZFs (i.e., where more than 10 amino acid residues separate two zinc finger structures) (Englbrecht et al., 2004). In animals and yeasts, the majority of C2H2-ZFPs belong to Classes A and B, whereas plant C2H2-ZFPs mainly belong to Class C. Based on the number of amino acid residues between the two conserved H residues of the ZF, Class C can be further divided into three subclasses: C1, C2, and C3, which contain 3, 4, and 5 residues, respectively. In subclass C1, 85% of C2H2-ZFPs contain the conserved motif QALGGH (or a new variant). In studies on rice C2H2-ZF genes, this type is also called the Q-type ZF (Englbrecht et al., 2004; Agarwal et al., 2007). Previous studies have identified 64 Q-type C2H2-ZF genes in *A. thaliana* genome (Englbrecht et al., 2004), 99 in rice (*Oryza sativa*) (Agarwal et al., 2007), and 47 in wheat (*Triticum aestivum*) (Kam et al., 2008), while in our study, 110 have been identified in maize.

Notably, when considered exclusively as a transcription factor, C2H2-ZFPs exhibit sequence-specific binding to DNA, whereas as a ZFP, these proteins can also identify RNA or other proteins (Gamsjaeger et al., 2007). However, researchers have primarily focused on their role as transcription factors, demonstrating their involvement in a wide range of physiological and biochemical processes, including development and organogenesis, along with stress response and defense pathways. In *Arabidopsis*, these proteins are involved in stress responses to salt, cold, drought, and light (Sakamoto et al., 2004; Davletova et al., 2005; Mittler et al., 2006; Ciftci-Yilmaz et al., 2007). In rice, they participate in vegetative growth, flower development, and drought stress resistance (Wu et al., 2008; Li et al., 2009). In *Petunia*, they are also related to flower development and drought stress resistance (Takatsuji et al., 1992; Sugano et al., 2003). In *Capsicum annuum*, they are involved in pathogen defense (Kim et al., 2004). In *Thellungiella halophila*, they are related to salt stress (Xu et al., 2007). Together, these studies suggest that the C2H2-ZF genes may not respond to a specific type of stress but can regulate multiple stress responses. Moreover, these known functions of C2H2-ZF genes account for only a very small part of their predicted repertoire of functions and thus are not sufficient to represent the functional properties of the entire C2H2-ZF gene family. Genomic sequencing work has demonstrated a large number of transcription factor family genes in the plant genome. The ever-increasing genomic sequencing information provides a wealth of information for analyzing the molecular evolutionary mechanisms of the C2H2-ZF gene family through bioinformatics and comparative genomics methods. At present, the evolutionary analysis of the C2H2-ZF family gene is mainly found in *Arabidopsis* and rice. On the other side, because our knowledge about C2H2-ZF genes’ function in maize is still limited. In addition to the essential role of the maize plants in molecular research as a model plant. Thus, we performed an evolution and functional prediction analysis of the maize C2H2-ZF genes. Moreover, the association analysis and the relationship between maize C2H2-ZF genes and other C2H2-ZF genes with known functions showed the essential role of maize genes, especially *ZmZFP126*. Thus, we select this gene for cloning and the discovery of favorable allelic variation. This characteristic of the *ZmZFP126* gene may be related to its location on the chromosome, specifically, near the centromere on maize chromosome 6, as the centromere region is considered to have a relatively low recombination rate. Finally, our analysis of the expression and functional properties of the C2H2-ZF family genes in the maize genome will contribute to a comprehensive understanding of the evolution of the C2H2-ZF domain.

## Materials and Methods

### Dataset

In this study, the data used included 20 adult-plant stage agronomic traits of 513 maize inbred lines (Yang et al., 2014), and genotype data corresponding to the population; 20 aboveground and root traits of maize seedlings of the same population under low phosphorus and normal phosphorus conditions were investigated (Zhang et al., 2014). The genotype data included 556,809 high-quality single nucleotide polymorphism (SNPs) markers (Minor Allele Frequency, MAF ≥0.05) (Fu et al., 2013; Li et al., 2013), downloaded from http://www.maizego.org/Resources.html.

### Identification of Maize ZFPs and Conserved ZFP-Associated Motifs

To identify the C2H2-ZFPs, we searched the *Zea mays* proteome (ftp://ftp.ensemblgenomes.org/pub/plants/release-29/fasta/zea_mays/pep/) using HMMER package 3.1b2 (Eddy, 1998) and the Pfam domain ZF-C2H2 (PF00096) (Bateman et al., 2004). The minimal cut-off score for the search was 0. The choice of this rather low threshold permits the detection of all ZFs/ZFPs and several false positives. The C2H2-ZFs and other conserved domains in the proteins coded by these genes were determined using SMART (http://smart.embl-heidelberg.de/) and InterProScan (http://www.ebi.ac.uk/interpro/search/sequence-search/), along with manual inspection.

### Phylogenetic Analysis of ZFPs

We extracted all the C2H2-ZF domains of maize ZFPs according to the SMART (http://smart.embl-heidelberg.de/)-identified C2H2-ZF motif sequence, a neighbor-joining (NJ) phylogenetic tree was also constructed (El-Sappah et al., 2021a). The Bootstrap test was repeated 1,000 times.

### Expression Analysis of C2H2-ZFPs in Maize

Microarray-based data analyses of both development and stress responses were performed to gain insight regarding the family-wide expression profile of C2H2-ZFPs and obtain the genes with highly significant differential expression. To analyze the spatial and temporal expression patterns of the *ZmC2H2* genes during development, transcriptome data of the genome-wide gene expression atlas of the maize inbred line B73, generated using NimbleGen microarray technology, were downloaded from Plexdb (ZM37) (http://www.plexdb.org/modules/PD_browse/experiment_browser.php?experiment=ZM37&genechip=nimbMaizeV2). Gene expression data from Agilent-025271 *Zea mays* Mais_array_v1 (GPL14913) under conditions of low T, N, and P were downloaded from Gene Expression Omnibus with accession numbers GSE46704 (Low P, T, N). In the preparatory stages of this study, our research team selected a drought-tolerant maize inbred line Ac7643 (P1) and a drought-sensitive inbred line Ac7729/TZSRW (P2) to simulate drought treatment and extract RNA. Two inbred maize lines were subjected to whole transcriptome sequencing (RNA-seq) using the Illumina HiSeq 2000 technology platform. We extracted the expression data of the C2H2-ZF gene from this data (RNA-seq), and used it to analyze the differential expression pattern of C2H2-ZF genes between different maize inbred lines under drought conditions. All microarray data were imported into R and Bioconductor (http://www.bioconductor.org/) for expression analyses. The ‘gplots’ package was then used to generate heatmaps.

### Candidate Gene-Based Association Mapping of the C2H2-ZF Genes in Maize

An association panel of 513 inbred maize lines was used to identify single nucleotide polymorphisms (SNPs) in the C2H2-ZF family genes significantly associated with 20 adult-plant stage agronomic traits and 19 shoot and root traits in the seeding stage under normal afforded and non-afforded phosphorus. This panel contained 556,809 high-quality SNPs (Fu et al., 2013; Li et al., 2013), downloaded fromhttp://www.maizego.org/Resources.html. The SNPs of the 247 C2H2-ZF genes were obtained from the panel using perl script. In order to eliminate the influence of environmental factors, best linear unbiased predictors were generated for each trait in different environments using SAS v8 package (SAS Institute Inc., Cary, NC, USA). The general linear model (GLM) and GLM combined with Q matrix (population structure) in TASSEL v3.0 (Bradbury et al., 2007) were used to conduct association mapping between the SNPs of candidate genes with the 20 traits in the 513 maize inbred lines, with *p* < 0.0001 being considered the significant threshold.

### The Relationship Between Maize and Other C2H2-ZF Genes With Known Functions

NCBI PubMed literature database (https://pubmed.ncbi.nlm.nih.gov/32061337/) has been used to find the protein sequences of C2H2-ZF genes with known function for constructing NJ phylogenetic tree with the maize C2H2-ZF genes. Furthermore, the iTOL online server (https://itol.embl.de/) has been used for further beautify.

### *ZmZFP126* Gene Cloning and Discovery of Favorable Allelic Variation

The modified CTAB method (Porebski et al., 1997) extracted genomic DNA of 109 inbred maize lines from different countries and regions (Supplementary Table S1). Using the Primer-BLAST program of NCBI’s BLAST tool (https://blast.ncbi.nlm.nih.gov/Blast.cgi), specific primers were designed using the DNA sequence of the first transcript of *ZmZFP126* gene (4,516 bp) as a template (Table 1). Using specific primers and based on TaKaRa’s high-fidelity PCR amplification enzyme, *ZmZFP126* gene DNAs of 109 inbred maize lines were amplified and sequenced. The obtained sequences were subject to multiple sequence alignment analysis with MAFFT software. Principal component analysis (PCA) was performed on the genotypes of 109 inbred maize lines using R software. SNPs and Indels were extracted from the *ZmZFP126* gene sequence using TASSEL v3.0 software, and the general linear model (GLM) combined with PCA was selected to perform correlation analysis on SNPs and Indels of *ZmZFP126* gene and 20 agronomic traits of 109 inbred maize lines. At the same time, the linkage disequilibrium (LD) level of the *ZmZFP126* gene in maize was analyzed using TASSEL v3.0 software.

**TABLE 1:** types of zinc-finger motifs in maize.

Type of finger		Conserved motif sequence^a^	Number of fingers	Conserved spacing^b^
**Q**		QALGGH	147	CX(2)CX(12)HX(2, 3, 4)H
**M**	M1	ALGGH	8	CX(2)CX(12)HX(3)H
	M2	Q_LGGH	4	CX(2)CX(12)HX(3)H
	M3	LGGH	6	CX(2)CX(12)HX(3)H
	M4	QALG_H	7	CX(2)CX(11, 12)HX(2, 3, 4)H
	M5	QA_GGH	1	CX(2)CX(12)HX(3)H
	M6	AL_GH	5	CX(2)CX(12)HX(3)H
	M7	L_GH	3	CX(1, 2)CX(12, 17)HX(3, 4, 6)H
	M8	A_ _GH	4	CX(2)CX(12)HX(3)H
	M9	ALG_H	2	CX(2)CX(12)HX(3, 4)H
	M10	LG_H	6	CX(2, 4)CX(12)HX(3, 4)H
	M11	Q_LG_H	1	CX(2)CX(12)HX(3)H
**Z**	Z1	NKKFKSDKQWKNHEQSKKH	5	CX(2)CX(12)HX(5)H
	Z2A	GFQR_QNLQ_HRR_H	22	CX(2)CX(12)HX(3)H
	Z2B	KTFNR_NNMQMHMWGH	9	CX(2)CX(12)HX(3)H
	Z2C	GKGF_RDANLRMHMR_H	5	CX(2)CX(12)HX(3)H
	Z3A	HH_ _ _ _ALGDL_GIKKH_ _RKH	21	CX(4)CX(17)HX(4)H
	Z3B	RN_ _ _HPRARPLKDFRTLQTHY_RKH	10	CX(3)CX(20)HX(4)H
	Z3C	KRNK_H_ _FQPLKTILCVKNHYK_SH	4	CX(4)CX(20)HX(4)H
	Z4A	K_YAV_SD_KAHLKTCGTRGH	5	CX(2)CX(12)HX(8)H
	Z4B	K_YAV_ _D_KAH_K	16	CX(2)CX(12)HX(2)
	Z4C	K_FS_ _ADL_THEKH	4	CX(2)CX(13)HX(2)H
	Z4D	K_FAV_GDWRTHEK	10	CX(2)CX(12)HX(2, 5)
	Z5A	GSEFKHKRSLKDHARAFGH	2	CX(1)CX(12)HX(5)H
	Z5B	GS_FKHKRSL_DH_R_FG	8	CX(1)CX(12)HX(4,5)
	Z6	VVRSKKCL_ _AH	4	CX(3)CX(12)HX(3)H
	Z7	S_ _G_ _ _EL_KH	4	CX(4)CX(11)HX(4)H
	Z8	TKLFHA_EFV_KH_ _LKH	4	CX(4)CX(12)HX(4)H
	Z9	DKAYIH_YKLNLHLK_ _H	3	CX(4)CX(12)HX(4)H
	Z10	K_F_ _ _SKLK_H	5	CX(4)CX(12)HX(3)H
	Z11	G_ _F_K_AHLKQHMQSH	4	CX(2)CX(12)HX(3)H
	Z12	G_AFSLDFNL_ _H	3	CX(4)CX(12)HX(3)H
	Z13	F_ _VSD_ _RH	4	CX(4)CX(12)HX(2,4)H/-
**C**			126	CX(1, 2, 4)CX(10, 11, 12, 13, 14, 15, 16, 17)
				HX(1, 2, 3, 4, 5, 6, 7)H

a ‘_’ represent the amino acid of deletion.

b The letters C, H, and X represent cysteine, histidine, and any amino acid, respectively; the numeral in brackets represents the number of amino acids.

## Results

### Identification of Maize ZFPs and Conserved ZFP-Associated Motifs

Three hundred twenty-six sequences from maize proteome version 2 were searched using HMMER and manual inspection, out of which 203 were unique, whereas the remaining 123 belonged to different transcripts of 44 genes (Supplementary Table S2). The SMART analysis of these 326 sequences revealed that all contained ZF domain(s). Some proteins were also found to contain KOW, DnaJ, SET, CactinC_cactus, Kin17_mid, C3H-ZF, U1-ZF, RING finger, Act-Frag_cataly, Amino_oxidase, transmembrane region, CMAS, RPT, SCOP, ANK, SEC14, EGF, G-patch, CHROMO, DSPc, F420_oxidored, SANT, IPPT, Di19_C, DUF3546, LYAR-ZF, AN1-ZF, Jmj, EXO3, DFD1, DUF1644, ARS2, Telomere_Sde2_2, PUG, SET, SF3a60_bindingd, and UBA domains as determined using the SMART analysis.

### Types of ZFs in Maize and Their Evolutionary Relationship

Four hundred and seventy-two individual ZFs were found in the 247 C2H2-ZFPs. Two extremely different kinds of motif, bearing no sequence similarity except for the presence of two C and two H residues, were observed (Table 2). The first type contained a conserved sequence “QALGGH” in the DNA-recognition motif and was designated as Q-type. This sequence has been shown to be specific to plants (Takatsuji et al., 1994), and 147 such ZFs have been found in maize. The other distinct kind of ZF was designated as C-type, as these did not contain any conserved motif in the ZF region. Certain modifications observed in the Q-type ZFs were classified as M-type and were numbered from M1-M11, indicating the type of modification as shown in Table 2. The M-type fingers have also been found in petunia (Kubo et al., 1998), *Arabidopsis* (Englbrecht et al., 2004), and rice (Agarwal et al., 2007). Among the C-type ZFs, some contained highly conserved motifs in the finger and flanking regions and were designated as Z-type; the 12 variants of Z-type were designated Z1-Z12 (Table 2).

**TABLE 2:** The association mapping of SNPs and Indels of *ZmZFP126* genes with 19 shoot and root traits in the maize seeding stage under normal afforded phosphorus (AP) and non-afforded phosphorus conditions (NAP).

Trait	Marker^a^	Site	*p* Value	R^2^
RL1-NAP	chr6.S_0045	45	0.0043	0.08589
RSA1-NAP	chr6.S_0045	45	0.0053	0.08289
LRL-AP	chr6.S_0045	45	0.0068	0.07800
RV1-NAP	chr6.S_0045	45	0.0072	0.07799
RL1-NAP	chr6.S_0248	248	0.0043	0.08589
RSA1-NAP	chr6.S_0248	248	0.0053	0.08289
LRL-AP	chr6.S_0248	248	0.0068	0.07800
RV1-NAP	chr6.S_0248	248	0.0072	0.07799
TRL-AP	chr6.S_1734	1734	0.0052	0.08917
RF-AP	chr6.S_1734	1734	0.0080	0.08065
TRSA-AP	chr6.S_1734	1734	0.0081	0.08101
RL1-AP	chr6.S_1734	1734	0.0092	0.07765
RSA1-AP	chr6.S_1734	1734	0.0096	0.07855
TRL-AP	chr6.S_1737	1737	0.0058	0.08801
RF-AP	chr6.S_1737	1737	0.0087	0.07982
TRSA-AP	chr6.S_1737	1737	0.0089	0.07986
TRL-AP	chr6.S_1741	1741	0.0050	0.09077
TRSA-AP	chr6.S_1741	1741	0.0064	0.08649
RF-AP	chr6.S_1741	1741	0.0077	0.08217
RSA1-AP	chr6.S_1741	1741	0.0085	0.08187
RV1-AP	chr6.S_1741	1741	0.0089	0.08183
RL1-AP	chr6.S_1741	1741	0.0092	0.07858
RV2-AP	chr6.S_1741	1741	0.0098	0.07656
RSA2-AP	chr6.S_1741	1741	0.0099	0.07639
SDW-NAP	chr6.S_1750	1750	0.0090	0.08270
SDW-NAP	chr6.S_1800	1800	0.0090	0.08270
SDW-NAP	chr6.S_1881	1881	0.0090	0.08270
SDW-NAP	**chr6.S_2024**	2024	0.0090	0.08270
SDW-NAP	chr6.S_2056	2056	0.0090	0.08270
SDW-NAP	chr6.S_2114	2114	0.0090	0.08270
SDW-NAP	**chr6.S_2115**	2115	0.0090	0.08270
SDW-NAP	chr6.S_2142	2142	0.0090	0.08270
SDW-NAP	**chr6.S_2164**	2164	0.0090	0.08270
SDW-NAP	chr6.S_2170	2170	0.0090	0.08270
SDW-NAP	chr6.S_2175	2175	0.0090	0.08270
SDW-NAP	chr6.S_2287	2287	0.0050	0.09507
SDW-NAP	chr6.S_2295	2295	0.0090	0.08270
SDW-NAP	**chr6.S_2313**	2313	0.0090	0.08270
SDW-NAP	chr6.S_2329	2329	0.0090	0.08270
SDW-NAP	**chr6.S_2340**	2340	0.0090	0.08270
SDW-NAP	chr6.S_2361	2361	0.0090	0.08270
SDW-NAP	chr6.S_2369	2369	0.0090	0.08270
SDW-NAP	chr6.S_2395	2395	0.0090	0.08270
SDW-NAP	chr6.S_2431	2431	0.0090	0.08270
SDW-NAP	chr6.S_2434	2434	0.0090	0.08270
SDW-NAP	chr6.S_2456	2456	0.0005	0.14581
PH-NAP	chr6.S_2456	2456	0.0019	0.11751
TFW-AP	chr6.S_2456	2456	0.0019	0.11567
TFW-NAP	chr6.S_2456	2456	0.0023	0.11042
SDW-AP	chr6.S_2456	2456	0.0056	0.09553
TDW-NAP	chr6.S_2456	2456	0.0071	0.08912
SDW-NAP	chr6.S_2545	2545	0.0017	0.11759
TDW-NAP	chr6.S_2545	2545	0.0055	0.09338
SDW-NAP	chr6.S_2546	2546	0.0017	0.11759
TDW-NAP	chr6.S_2546	2546	0.0055	0.09338
SDW-NAP	**chr6.S_2580**	2580	0.0017	0.11759
TDW-NAP	**chr6.S_2580**	2580	0.0055	0.09338
SDW-NAP	chr6.S_2581	2581	0.0017	0.11759
TDW-NAP	chr6.S_2581	2581	0.0055	0.09338
SDW-NAP	chr6.S_2595	2595	0.0017	0.11759
TDW-NAP	chr6.S_2595	2595	0.0055	0.09338
SDW-NAP	chr6.S_2613	2613	0.0017	0.11759
TDW-NAP	chr6.S_2613	2613	0.0055	0.09338
SDW-NAP	chr6.S_2614	2614	0.0017	0.11759
TDW-NAP	chr6.S_2614	2614	0.0055	0.09338
SDW-NAP	chr6.S_2640	2640	0.0017	0.11759
TDW-NAP	chr6.S_2640	2640	0.0055	0.09338
LRL-NAP	chr6.S_2838	2838	0.0075	0.09024
RV1-AP	chr6.S_3501	3501	0.0038	0.10881
TRL-AP	chr6.S_3501	3501	0.0041	0.10461
RF-AP	chr6.S_3501	3501	0.0043	0.10473
RSA1-AP	chr6.S_3501	3501	0.0044	0.10494
TRSA-AP	chr6.S_3501	3501	0.0044	0.10221
RL1-AP	chr6.S_3501	3501	0.0071	0.09339

a Bold indicated Indel sites.

**FIGURE 1:**
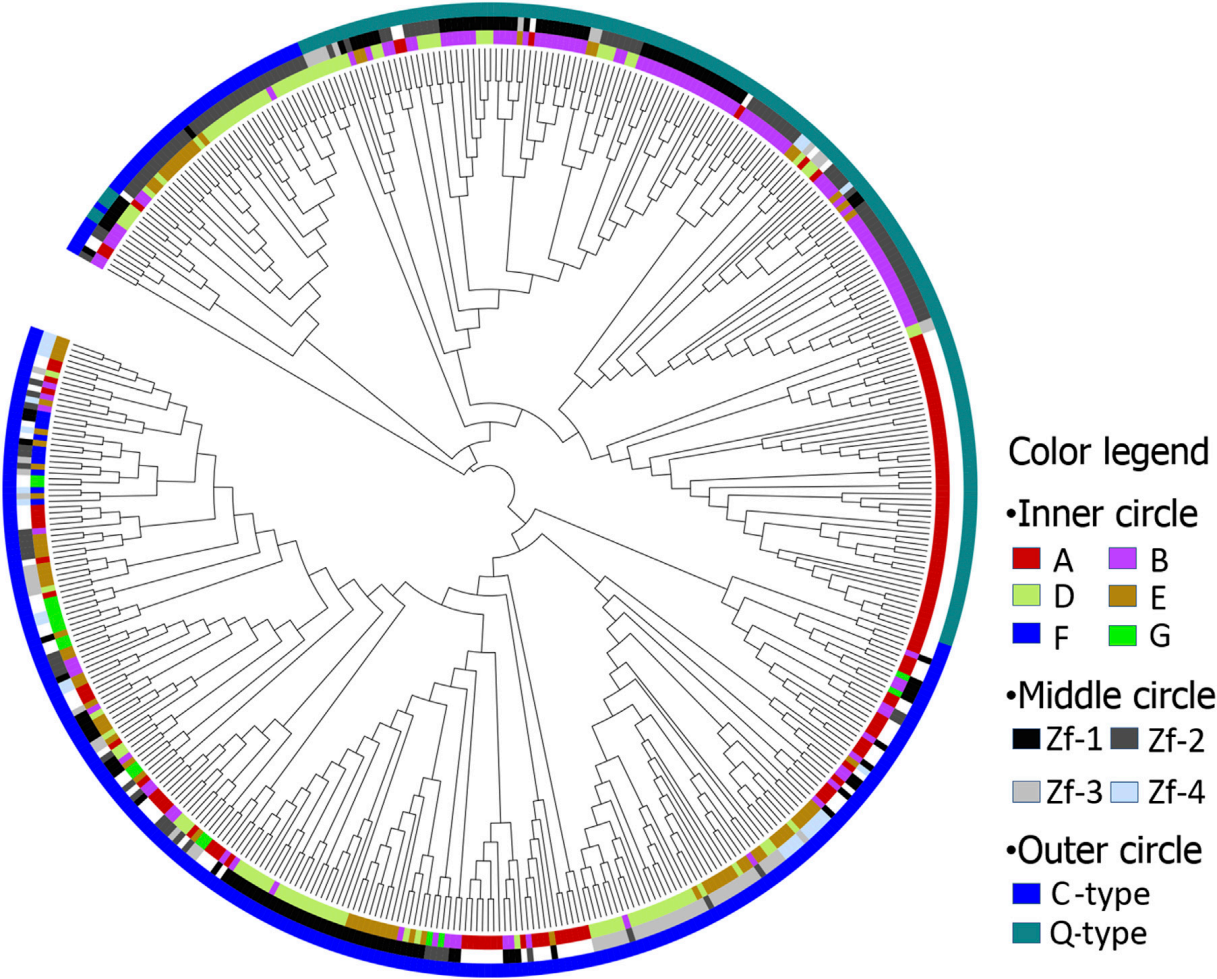
The phylogenetic tree of maize C2H2-ZF motif. Inner circle: A, B, D, E, F, and G represents ZF motifs in C2H2-ZF genes, including 1, 2, 3, 4, 5, and 9 ZF motifs, respectively; Middle circle: Zf-1, Zf-2, Zf-3, and Zf-4 represent first, second, third and fourth ZF motifs, respectively; Outer circle: C and Q represents C-type and Q-type ZF motifs, respectively.

An unrooted NJ-plotted phylogenetic tree was constructed using the 472ZFs domains sequences (Figure 1). This revealed that the following. 1) Maize C2H2-ZFs can be classified into two primary types, namely C-type and Q-type, of which the C-type was divided into two discontinuous groups (Figure 1, outer circle). 2) The position and order of ZFs appeared critical for genes containing multiple C2H2-ZFs, as numerous C2H2-ZF genes remained unchanged throughout evolution. Therefore, the ZFs exhibiting the same position and order were classified together (Figure 1, middle and inner circles). For example, the Q-type C2H2-ZF mainly comprises two kinds of ZF genes, those having a single (A) or two (B) ZFs, with the first and second ZFs of B genes being classified together. In addition, the first, second, and third ZFs of group D and E genes, containing three and four ZFs, respectively, were classified together.

### Expression of the C2H2-ZF Genes in Specific Organs During the Whole Maize Growing Period

Two hundred and twenty-eight transcripts of the C2H2-ZF domain-containing proteins were detected, belonging to 168 corresponding C2H2-ZF genes., Hierarchical clustering was performed to visualize the global transcription profile of the C2H2-ZF genes across the 11 maize organs and to understand the temporal and spatial transcription patterns in different developmental stages. As illustrated in Supplementary Figure S1, the resultant heatmap could be divided into three clusters. Clusters 1, 2, and 3 comprise 92, 39, and 38 members, respectively. Notably, the *ZmZFP236* gene has six transcripts, of which transcripts 1, 2, 3, and 4 belong to cluster 2, whereas transcripts 5 and 6 belong to cluster 3. Genes in cluster 1 exhibit relatively low expression, with the mean of the log-signal values of each gene ranging from 6 to 8. In contrast, cluster 3 contains genes with relatively high expression, with the mean of the log-signal values of each gene within the range of 12–14. In cluster 2, genes are expressed over a large range (from 6 to 14). Furthermore, 33.3 and 39.5% of genes in clusters 2 and 3, respectively, produce at least two transcripts, whereas only 5.5% of cluster 1 genes generate multiple transcripts.

To identify putative differentially expressed genes in specific organs or stages, we calculated the coefficient of variation (CV value; CV = SD/mean; where, SD represents the standard deviation and mean represents the mean expression level of each gene across all the tissues in the three clusters) to compare the degree of variation of each gene among distinct organs (Wei K.-F. et al., 2012; Wei K. et al., 2012) (Supplementary Table S3). The results showed that the CV of the expression of these transcripts in 11 maize tissues or organs exhibited a large range, from 1.4 to 39.9%. The gene with the lowest CV was *ZmZFP195*, belonging to cluster 3, highly expressed in the 11 maize tissues or organs (mean = 13.847), indicating that this gene may be a housekeeping gene in maize. To the best of our knowledge, there are no other reports of this gene in maize.

The CVs of cluster 3 were the smallest (1.4–13.2%), with the CV of 72% genes 46) being <5%. The results indicated that most of the genes in this group exhibited consistent expression patterns relative to those other C2H2-ZF genes. The gene with the highest expression variation in this group was *ZmZFP033* (*ZmZFP033.1*, *ZmZFP033.2*, and *ZmZFP033.3*) with a CV of 13.2%. This gene showed low expression in filaments (R1_Silks) but was highly expressed in most other tissues or organs, especially at 12–24 days after-pollination (DAP) in the whole seed and endosperm. In cluster 2, the CVs of 30% of the genes (20) were >17%, with the CVs of 15 genes exceeding 20%, including the *ZmZFP056* gene with the highest CV. The results indicated that most of the genes exhibit tissue-specific expression in this group. In particular, the expression CVs were 20 and 22% for the *ZmZFP144* and *ZmZFP178* (*ZmZFP178.1* and *ZmZFP178.2*) genes, respectively, which are highly expressed at 16–24 DAP in embryos and immature cobs. In turn, the *ZmZFP098* gene was highly expressed in the leaves of maize during the whole growth period, having an expression CV of 19%, suggesting that the gene might be involved in the development of leaf or response to light. The *ZmZFP152* gene, highly expressed at 2–24 DAP in the whole seed, innermost husk, and pericarp, had an expression CV of 18%, indicating that the gene is involved in the development of grain. The *ZmZFP142* and *ZmZFP166* genes had the same CV (20%) and were highly expressed in the leaves and anthers (R1_Anthers), and the gene structure and protein domains of these two genes are very similar; they belong to group III. The results suggested that these two genes perform the same function and may regulate reproductive growth.

The CVs of gene expression in cluster 1 were relatively stable compared with those in cluster 2, with expression CVs of 60% of the genes (59) being <10%, 19% of the genes 19) > 15%, and 7% genes 7) > 20%. The CVs of the *ZmZFP035*, *ZmZFP128*, and *ZmZFP209* genes were 21, 23, and 18%, respectively. These three genes were highly expressed in the anther (R1_Anthers). In turn, the CVs of genes *ZmZFP247* and *ZmZFP074* (*ZmZFP074.1*, *ZmZFP074.2*, and *ZmZFP074.3*) were 20 and 19.7%, respectively, both of which were highly expressed in meiotic tassels (V18_Meiotic tassel) and exhibited low expression in other tissues or organs. The results indicated that these two genes are closely related to tassel development.

### Expression of the C2H2-ZF Genes Under Abiotic Stress

**FIGURE 2:**
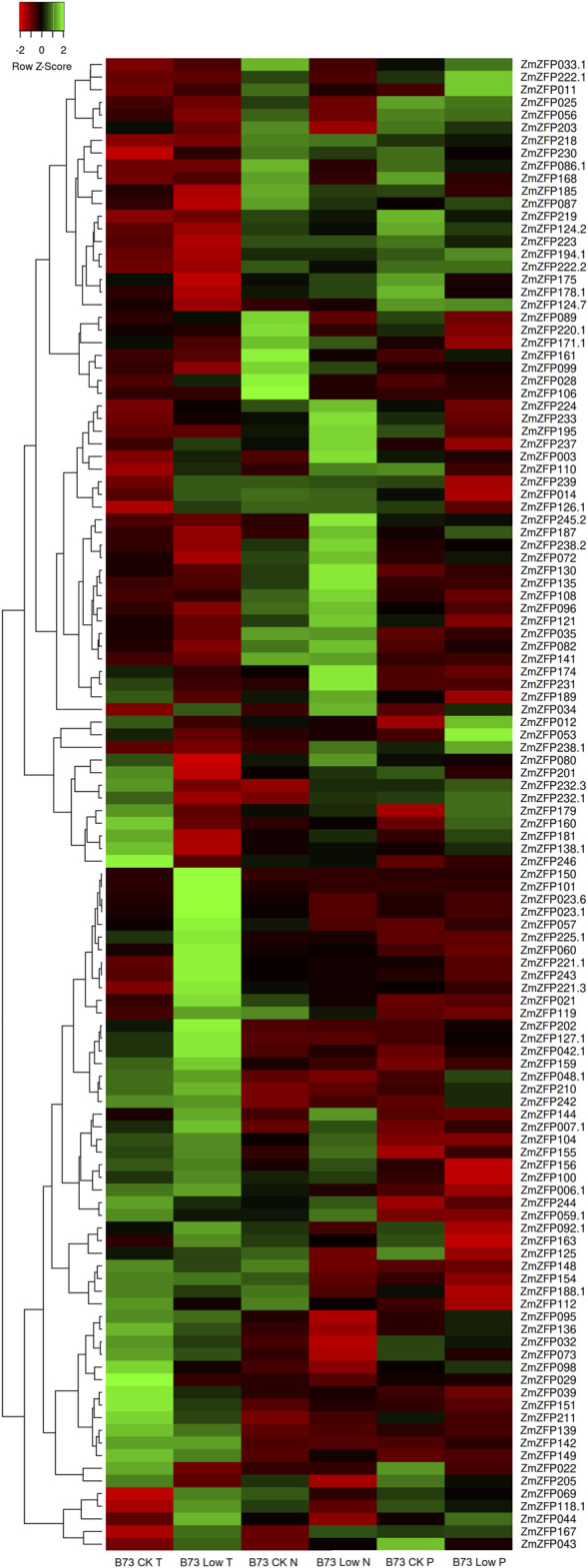
Expression profiles of maize C2H2-ZF genes under low temperature, low nitrogen, and low phosphorus. The green, red, and black represent positive, negative, and neutral gene expression levels, respectively. Heat map hierarchical clustering of log_2_-transformed genes under low temperature, low nitrogen, and low phosphorus.

As a ubiquitous transcription factor, the C2H2-ZF genes can regulate the expression of genes related to broad-spectrum stress. One hundred and eighteen probes were found to match 118 transcripts of 112 C2H2-ZF genes in the GPL14913 gene chip (Supplementary Table S4). The log_2_ (treated/control) ratio was illustrated using a heatmap (Figure 2), showing the fold change of each maize C2H2-ZF gene under low temperature (T), nitrogen (N), and phosphorus (P) conditions compared with the control (Figure 2; Supplementary Table S5). Under low T, N, and P conditions, the expression of 15, 10, and 8 genes changed by more than 2-fold (| Log_2_ (treated/control) |> 1) compared with that of the control (Supplementary Table S5). The maize C2H2-ZF genes exhibited different response patterns to different stress conditions, such as downregulated expression of the *ZmZFP106* gene under low N conditions, whereas no expression changes under low T and low P conditions. The response only to low N stress suggested that the gene may play an important negative regulatory role under low N conditions. In turn, the *ZmZFP072* and *ZmZFP181* genes were downregulated only under low T conditions but not under low N and low P conditions, which indicated that the two genes were mainly responsive to low T stress and had negative regulatory effects. The expression of the *ZmZFP101* and *ZmZFP150* genes was significantly upregulated under low T conditions; however, the *ZmZFP150* gene expression was not changed under low N and low P conditions, whereas *ZmZFP101* expression was down-regulated under low N condition, but not under low P condition. The results indicated that the *ZmZFP101* and *ZmZFP150* genes exhibit co-expression characteristics under low T conditions and stable low expression in various tissues or organs throughout the growth period of maize. The expression patterns of *ZmZFP160* and *ZmZFP053* showed similar responses under low T conditions, whereas the expression of both was significantly higher under low P conditions than that under low N conditions. These genes exhibit relatively stable and low expression in maize tissues or organs (Supplementary Figure S1). Notably, 25 maize C2H2-ZF genes exhibited > 2-fold changes in expression under low T, low N, and low P conditions, and 17 of which belonged to cluster 1 and showed stable expression in all tissues or organs at maize developmental stages (Supplementary Figure S1: Cluster 1). This indicated that these genes likely only play a role under stress conditions. In addition, four genes (*ZmZFP023*, *ZmZFP056*, *ZmZFP144*, and *ZmZFP178*) belong to cluster 2, of which *ZmZFP056* was highly expressed at 12–24 DAP the whole seeds and endosperm but also exhibited negative regulation under low N stress. *ZmZFP060*, belonging to cluster 3, showed stable and high expression in various tissues or organs at maize development stages, exhibited considerably decreased expression under low N stress and appeared to play a positive regulatory role under low T conditions.

**FIGURE 3:**
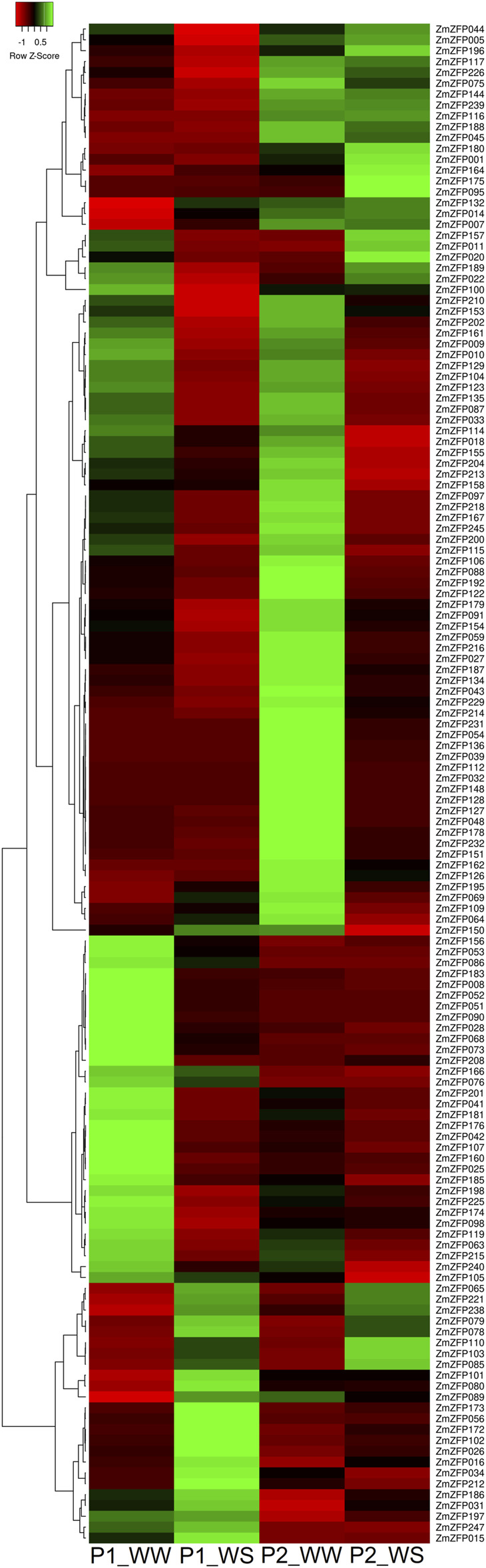
Expression profiles of Ac7643 inbred line (drought tolerance) and P2 inbred line (drought sensitive) C2H2-ZF genes under water-stressed conditions. The green, red, and black represent positive, negative, and zero gene expression levels, respectively. Heat map hierarchical clustering of log_2_ transformed gene under water-stressed. P1_WW: expression of Ac7643 inbred line under well-watered conditions; P1_WS: expression of Ac7643 inbred line under water-stressed conditions. P2_WW: expression of Ac7729/TZSRW inbred line under well-watered conditions; P2_WS: expression of Ac7729/TZSRW inbred line under water-stressed conditions.

A drought-sensitive maize inbred line P2 (Ac7729 × TZSRW) and drought-tolerant maize inbred line P1 (Ac7643) were used to investigate the effect of maize C2H2-ZF genes on drought stress resistance. The RNA of these two inbred lines under simulated drought conditions was extracted and sequenced. One hundred and ninety maize C2H2-ZF genes were detected from the RNA sequencing data (Supplementary Table S6). The other 50 C2H2-ZF genes were not detected in either inbred strain upon treatment or control, although the expression of 32 of these C2H2-ZF genes had been detected in the whole growth stage of maize (Supplementary Figure S1; Supplementary Table S3). We speculated that these 32 genes might be expressed under specific conditions and specific tissues. In P1 and P2 inbred lines under drought conditions, we identified 63 and 54 C2H2-ZF genes with 2-fold downregulated expression and 18 and 17 genes with >2-fold downregulated expression (Figure 3; Supplementary Table S6). The results showed that the C2H2-ZF genes were mainly subjected to negative regulation under drought stress, although some showed positive regulation effects. The number of C2H2-ZF genes expressed was significantly increased under drought stress compared with that under low T, N, and P stress (Supplementary Table S5; Supplementary Table S6), indicating that drought regulation is a more complex system that requires the involvement of more genes, and also revealing that the C2H2-ZF genes play an important role in regulating drought responses in maize.

### Candidate Gene-Based Association Mapping of the C2H2-ZF Genes in Maize

A total of 2998 SNPs of 159 C2H2-ZF genes were extracted from an association panel of 513 inbred maize lines, which contained 556,809 high-quality SNPs. Of these, 664 SNPs of 107 C2H2-ZF genes were significantly associated with 20 adult-plant stage agronomic traits (Supplementary Table S7). Specifically, 56% C2H2-ZF genes significantly associated with seed traits (e.g., 100-grain weight, kernel width, kernel length, and kernel thickness), 47.7% significantly associated with ear traits (e.g., ear diameter, ear length, ear row number, and kernel number per-row), 32.7 and 17.8% significantly associated with plant height and ear height, respectively, and 16.8% significantly associated with flowering traits (e.g., heading date, pollen shed, and silking time) (Supplementary Table S7). For example, all SNPs of the *ZmZFP077*, *ZmZFP091*, *ZmZFP110*, *ZmZFP138*, *ZmZFP150*, *ZmZFP144*, *ZmZFP169*, *ZmZFP183*, and *ZmZFP191* genes were significantly associated with seed traits. In most cases, the seed and ear traits were genetically linked. Most SNPs of the *ZmZFP024*, *ZmZFP064*, *ZmZFP095*, *ZmZFP227*, *ZmZFP124*, *ZmZFP238* and *ZmZFP242* genes were significantly associated with flowering traits. In addition, 241 SNPs of 60 C2H2-ZF genes were significantly associated with 19 shoot and root traits in the seeding stage under normal afforded (AP) and non-afforded P conditions (NAP) (Supplementary Table S8), of which 16 genes (e.g., *ZmZFP006*, *ZmZFP056*, *ZmZFP193*, and *ZmZFP202*) were only significantly associated with the shoot and root traits under NAP, whereas 27 were associated under both AP and NAP (Supplementary Table S8). Moreover, every SNP (except chr6: 39893953 site) of the *ZmZFP126* gene was significantly associated with at least four traits, and even the chr6: 39894469 sites was significantly associated with 24 traits under normal afforded and NAP.

### The Relationship Between Maize C2H2-ZF Genes and Other C2H2-ZF Genes With Known Functions

**FIGURE 4:**
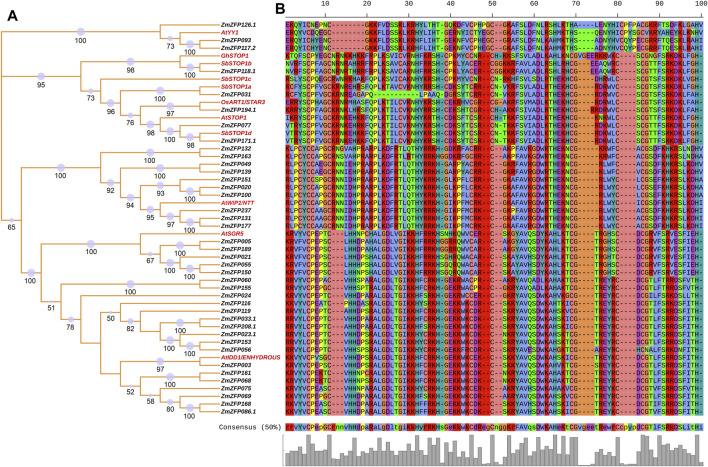
The relationship between maize C2H2-ZF genes and other C2H2-ZF genes with known functions. **(A)**: NJ phylogenetic tree of the relationships between the 11 C2H2-ZF genes with known functions and 40 maize C2H2-ZF genes. The red labels indicated the gene with known function; The number on the branch indicated the bootstrap value, and the size of the purple circle also indicated the size of the bootstrap value (bootstrap >50). **(B)**: The partial conserved amino acid motifs of these homologous genes.

Sixty-seven C2H2-ZF genes with known functions (Supplementary Table S9) have been reported to date, of which 66 have homologous genes in maize. Figure 4 shows the relationships between the 11 C2H2-ZF genes with known functions and 40 maize C2H2-ZF genes. The *AtYY1* gene in *Arabidopsis* (Li et al., 2016) has three homologous genes, namely, *ZmZFP126*, *ZmZFP093*, and *ZmZFP117*. The *AtYY1* gene is a novel negative regulator of the Arabidopsis ABA response network. Association analysis results showed that the maize *ZmZFP126* gene has four SNP loci (chr6.S_39893949, chr6.S_39893950, chr6.S_39893951, and chr6.S_39893952) that are associated with 100-grain weight, two SNP loci (chr6.S_39893951 and chr6.S_39894217) that are significantly associated with kernel length, and one SNP locus (chr6.S_39894034) that are significantly associated with tassel main axis length. In addition, this gene has 13 SNP sites that are simultaneously significantly associated with multiple root traits under NAP, such as the root forks under NAP (RF-NAP), root surface area 1 (the average root surface area in diameter between 0.0–0.5 mm of five plants) under NAP (RSA1-NAP). The maize *ZmZFP117* gene has two SNP loci (chr5.S_172019275 and chr5.S_172019305) that are significantly associated with leaf number above the ear. In addition, one SNP (SYN19115) is significantly associated with the root forks under AP (RF-AP), root length 1 (the average root length in diameter between 0.0–0.5 mm of five plants) under AP (RL1-AP), and total root length under AP (TRL-AP) traits. The functions of the *Arabidopsis AtSTOP1* gene (Iuchi et al., 2007; Sawaki et al., 2009), the *Gossypium hirsutum GhSTOP1* gene (Kundu et al., 2018), the rice *OsART1/STAR3* gene (Yamaji et al., 2009; Tsutsui et al., 2011), *and the sorghum SbSTOP1 genes* (*SbSTOP1a, SbSTOP1b, SbSTOP1c, and SbSTOP1d*) (Huang et al., 2018; Gao et al., 2019), are all associated with aluminum stress, and these genes are homologous to the maize *ZmZFP031*, *ZmZFP118.1*, *ZmZFP194.1*, *ZmZFP077*, and *ZmZFP171* genes. The maize *ZmZFP118* gene has four SNP loci (chr5.S_175685185, chr5.S_175689281, chr5.S_175689296, and chr5.S_175689575) that are significantly associated with kernel thickness and kernel number per-row and two SNP loci (chr5.S_175687470 and chr5.S_175688702) that are significantly associated with kernel length. In addition, two SNP loci of this gene are significantly associated with leaf number under NAP (LN-NAP), and four SNP loci are significantly associated with root volume 2 (the average root volume in diameter between 0.5–1.0 mm of five plants) under NAP (RV2-NAP). The maize *ZmZFP194* gene has three SNP loci (chr10.S_10140760, chr10.S_10140901, and chr10.S_10140902) that are significantly associated with ear leaf width. The chr10.S_10140760 locus is also significantly associated with the ear diameter. Two SNP loci (chr10.S_10140916 and chr10.S_10143604) are significantly associated with leaf number above the ear, and the chr10.S_10142324 SNP locus is significantly associated with kernel length. In addition, the chr10.S_10136241 SNP locus of this gene is significantly associated with multiple seedling traits in maize under NAP (e.g., RDW-NAP, RL2-NAP, RSA2-NAP, RV2-NAP, TDW-NAP, TFW-NAP, and TRV-NAP). The *Arabidopsis AtWIP2/NTT* gene is associated with fruit dehiscence and replum development (Chung et al., 2013; Marsch-Martínez et al., 2014), and this gene is homologous to the maize *ZmZFP132*, *ZmZFP163*, *ZmZFP049*, *ZmZFP139*, *ZmZFP151*, *ZmZFP020*, *ZmZFP100*, *ZmZFP237*, *ZmZFP131*, and *ZmZFP177* genes. However, no SNP locus significantly associated with maize traits has been detected in these genes. The *Arabidopsis AtSGR5* gene (Morita et al., 2006; Kim et al., 2015) is involved in early events of gravitropism in *Arabidopsis* inflorescence stems. This gene is homologous to the maize *ZmZFP005*, *ZmZFP189*, *ZmZFP021*, *ZmZFP055*, and *ZmZFP150* genes. The SNP locus chr9.S_144035764 in the maize *ZmZFP189* gene is significantly associated with ear leaf length. The *ZmZFP150* gene has two SNP loci (chr7.S_124146886 and SYN38697) that are also significantly associated with kernel length. The *Arabidopsis AtIDD1/ENHYDROUS* gene promotes the transition to germination by regulating light and hormonal signaling during seed maturation (Feurtado et al., 2011; Fukazawa et al., 2014). This gene is homologous to 17 maize C2H2-ZF genes (Figure 4). The *ZmZFP060* gene has four SNP loci (chr2.S_207380320, chr2.S_207381507, chr2.S_207381508, and chr2.S_207381537) that are significantly associated with kernel length. Three among them (chr2.S_207381507, chr2.S_207381508, and chr2.S_207381537) are simultaneously significantly associated with ear length. The chr2.S_207380848 and chr2.S_207380851 loci of this gene are significantly associated with 100-grain weight, while the chr2.S_207380910 and chr2.S_207380911 loci of the same gene are also significantly associated with cob diameter and ear diameter. Four SNPs (chr2.S_207378625, chr2.S_207378663, chr2.S_207380989, and chr2.S_207381192) are significantly associated with tassel branch number. The chr2.S_207379647 and chr2.S_207379650 loci are significantly associated with plant height. Four SNP loci of the *ZmZFP155* gene (chr7.S_160601597, chr7.S_160601615, chr7.S_160601621, and chr7.S_160601626) are significantly associated with three flowering traits: heading date, pollen shed, and silking time. The chr7.S_160601466, chr7.S_160601469, chr7.S_160601395, and chr7.S_160600519 loci are significantly associated with kernel number per-row. This gene has seven SNP loci (chr7.S_160598451, chr7.S_160599436, chr7.S_160599437, chr7.S_160599580, chr7.S_160600407, chr7.S_160600877, and chr7.S_160601398) that are significantly associated with kernel length. The chr7.S_160600894 SNP locus of the gene is significantly associated with LN-NAP, and the chr7.S_160600586 and chr7.S_160600590 loci are significantly associated with the longest root length under AP (LRL-AP). In the *ZmZFP024* gene, three SNP loci (SYN38850, SYN38852, and PZB02144.4) are significantly associated with silking time. In the *ZmZFP119* gene, 27 SNPs are significantly associated with ear leaf length, ear length, kernel length, plant height, and tassel main axis length, and 13 of these are significantly associated with kernel length. In addition, the gene has 14 SNP loci that are significantly associated with seedling traits under AP, of which three SNP loci (chr5.S_194170562, chr5.S_194170563, and chr5.S_194170564) are significantly associated with root/shoot ratio under AP (R/S-AP). In the *ZmZFP033* gene, seven SNP loci are significantly associated with kernel width. The chr2.S_17095383 locus is also significantly associated with 100-grain weight and ear diameter. In addition, the gene has four SNP loci that are significantly associated with seedling traits under NAP. In the *ZmZFP208* gene, 22 SNP loci are more associated with 100-grain weight, ear row number, kernel thickness, kernel width, kernel length, and other grain-related traits. This gene has four SNP loci more associated with root dry weight under AP and NAP (RDW-AP and RDW-NAP). Three SNP loci are significantly associated with R/S-AP. In the *ZmZFP023* gene, three SNPs (chr1.S_206123397, chr1.S_206123974, and SYN14326) are significantly associated with cob weight, ear length, and ear diameter in maize, respectively. Four SNP loci in the *ZmZFP153* gene are significantly associated with grain traits (kernel thickness, ear row number, kernel width, and kernel thickness). In addition, the gene has two SNP loci that are significantly associated with three NAP traits: shoot dry weight under NAP (SDW-NAP), total fresh weight NAP (TFW-NAP), and plant height under NAP (PH-NAP). The nine SNP loci in the *ZmZFP056* gene are all significantly associated with tassel branch number. Among them, the chr2.S_197936812 locus is also significantly associated with kernel width. In addition, this gene has 18 SNP loci that are significantly associated with multiple seedling traits under NAP, and each SNP locus is significantly correlated with LN-NAP. The *ZmZFP003* gene has five SNPs significantly associated with grain traits (e.g., kernel thickness and kernel length) and ear traits (ear diameter and ear length). One SNP locus in the *ZmZFP075* gene is significantly associated with kernel thickness. The chr3.S_5884603 locus of the *ZmZFP069* gene is associated primarily with flowering traits (heading date, pollen shed, and silking time), and the chr3.S_5886671 and chr3.S_5886865 loci are significantly associated with ear diameter. The chr3.S_5884749 locus of this gene is significantly associated with multiple seedling traits under AP. Eight SNPs in the *ZmZFP168* gene are significantly associated with kernel length. The chr8.S_139345593 locus is significantly associated with both ear length and kernel number per-row. The three SNP loci PZE-108082637, chr8.S_139345593, and chr8.S_139346331 are also significantly associated with root traits of maize seedlings under AP.

The *Zoysia japonica ZjZFN1* gene is involved in salt stress in *Arabidopsis* (Teng et al., 2018). This gene is homologous to the maize genes *ZmZFP026* and *ZmZFP105*. The association analysis results showed that the *ZmZFP026* gene has six SNP loci associated with female ear traits (e.g., cob diameter, ear diameter, cob weight, and ear length). In addition, the chr1.S_279912850 locus is significantly associated with the longest root length under NAP (LRL-NAP), and the chr1.S_279912890 locus is significantly associated with plant height under AP (PH-AP). The *ZmZFP105* gene has two SNP loci (chr5.S_6315680 and chr5.S_6316039) that are significantly associated with 100-grain weight, four SNP loci (chr5.S_6316055, chr5.S_6316450, chr5.S_6316451, and chr5.S_6316452) that are significantly associated with kernel length, and the chr5.S_6316039 and chr5.S_6316452 loci that are significantly associated with ear length and ear diameter, respectively. In addition, the chr5.S_6315637 locus is significantly associated with leaf number under AP (LN-AP). The *Arabidopsis AtCZS/SUVR5* gene encodes a histone methyltransferase that functions together with its binding partner LDL1/SWP1 as one of the regulators of flowering timing in *Arabidopsis* (Krichevsky et al., 2007; Grewal et al., 2012); it is homologous to three maize genes: *ZmZFP096*, *ZmZFP085*, and *ZmZFP120*. Five SNP loci in the *ZmZFP096* gene are significantly associated with plant height; two SNP loci with cob diameter; and one locus (chr4.S_160935992), with kernel length and kernel number per row. The chr5.S_201600785 locus of *ZmZFP120* is significantly associated with ear height and root diameter under NAP (RD-NAP). The rice *OsEMF2B* and *OsEMF2A* (Li et al., 2009; Luo et al., 2009), *Arabidopsis AtEMF2* (Yoshida et al., 2001), and cabbage *BoEMF2.1* and *BoEMF2.2* genes (Liu et al., 2012) regulate diverse genetic pathways and are required for vegetative development and plant survival. These genes are homologous to the maize *ZmZFP241* gene, and the chr7.S_84537039 locus of *ZmZFP241* is significantly associated with cob weight. The *Arabidopsis AtSE* gene controls leaf development, meristem activity, inflorescence architecture, and developmental phase transition (Prigge and Wagner, 2001; Yang et al., 2006). This gene has four homologous genes in maize, namely, *ZmZFP214*, *ZmZFP227*, *ZmZFP238*, and *ZmZFP234*. The *ZmZFP214* gene has four SNP loci that are significantly associated with tassel branch number and three SNP loci that are significantly associated with ear leaf width. In addition, this gene has two SNP loci that are significantly associated with root traits under NAP, and the other 12 SNP loci are significantly associated with root traits under AP. Seven SNPs in the *ZmZFP227* gene are simultaneously significantly associated with ear leaf length, heading date, plant height, pollen shed, and silking time. The chr4.S_42145910 and chr4.S_42145955 loci are also significantly associated with the flowering traits of pollen shed and silking time. Eight SNP loci are significantly associated with kernel length, and the chr4.S_42148067 and chr4.S_42148068 loci are significantly associated with kernel thickness. The *ZmZFP238* gene has six SNP loci that are significantly associated with silking time and two SNP loci that are significantly associated with kernel length. The *Arabidopsis AtJAG* (Dinneny et al., 2004; Ohno et al., 2004; Dinneny et al., 2006) and *AtJGL* (Dinneny et al., 2006) and rice *OsSL1*(Xiao et al., 2009) genes are homologous to the maize *ZmZFP159* gene. *AtJAG* and *AtJGL* define stamen and carpel shape in *Arabidopsis*, and *OsSL1* regulates floral organ identity in rice. The association analysis results showed that the PZE-108011044 locus of the *ZmZFP159* gene is significantly associated with kernel number per row, and the PZE-108011046 locus is significantly associated with plant height and tassel main axis length.

*ZmMRPI-1 (ZmZFP174) and ZmMRPI-2* (*ZmZFP080*) (Royo et al., 2009) interact with *ZmMRP-1* and modulate its activity on transfer cell-specific promoters. The results of association analysis showed that the chr8.S_171810459 and chr8.S_171805538 loci of the *ZmZFP174* gene are significantly associated with the kernel width and RT2-AP traits, respectively. The chr3.S_190064606 locus of the *ZmZFP080* gene is significantly associated with kernel thickness, and the chr3.S_190065192 locus is significantly associated with multiple root traits under NAP. The *Arabidopsis AtSUF4* (Resentini et al., 2017) gene supports gamete fusion via regulating *Arabidopsis EC1* gene expression. This gene is homologous to the *ZmZFP058* gene, and the chr2.S_198580873 locus of the *ZmZFP058* gene is significantly associated with multiple traits, including heading date, pollen shed, silking time, kernel length, leaf number above the ear, ear leaf length, ear length, and plant height. The chr2.S_198582212 and chr2.S_198582325 loci are significantly associated with LN-NAP. The *Arabidopsis SUPERMAN/FLO10/SUP* gene is required for maintaining the boundaries between stamens and carpels and for regulating the development of the outer ovule integument (Sakai et al., 1995; Yun et al., 2002; Isernia et al., 2003; Ito et al., 2003; Prunet et al., 2017; Xu et al., 2018). The cucumber *SUPERMAN* gene has a conserved function in stamen and fruit development and a distinct role in floral patterning (Wu et al., 2014). The petunia *PhSUP1* gene plays a distinct role in floral organ morphogenesis (Nakagawa et al., 2004), and the *SlSUP* gene has a positive role in the female flower developmental pathways of *S*. *latifolia* (Kazama et al., 2009). These genes are homologous to the maize *ZmZFP087* gene, and the results of association analysis showed that the chr4.S_610398 locus of the *ZmZFP087* gene is significantly associated with cob weight. The *Arabidopsis AtZFP8* gene plays partially redundant and essential roles in inflorescence trichome initiation and its regulation by gibberellic acid and cytokinins (Gan et al., 2007). This gene is homologous to the maize *ZmZFP122* gene, and the chr5.S_211549650 and chr5.S_211549874 loci of the *ZmZFP122* gene are significantly associated with pollen shed and ear row number, respectively. In addition, the chr5.S_211549697 and chr5.S_211549790 loci of this gene are significantly associated with multiple maize seedling traits under AP.

### Mining of Favorable Allelic Variants of *ZmZFP126* Gene

**FIGURE 5:**
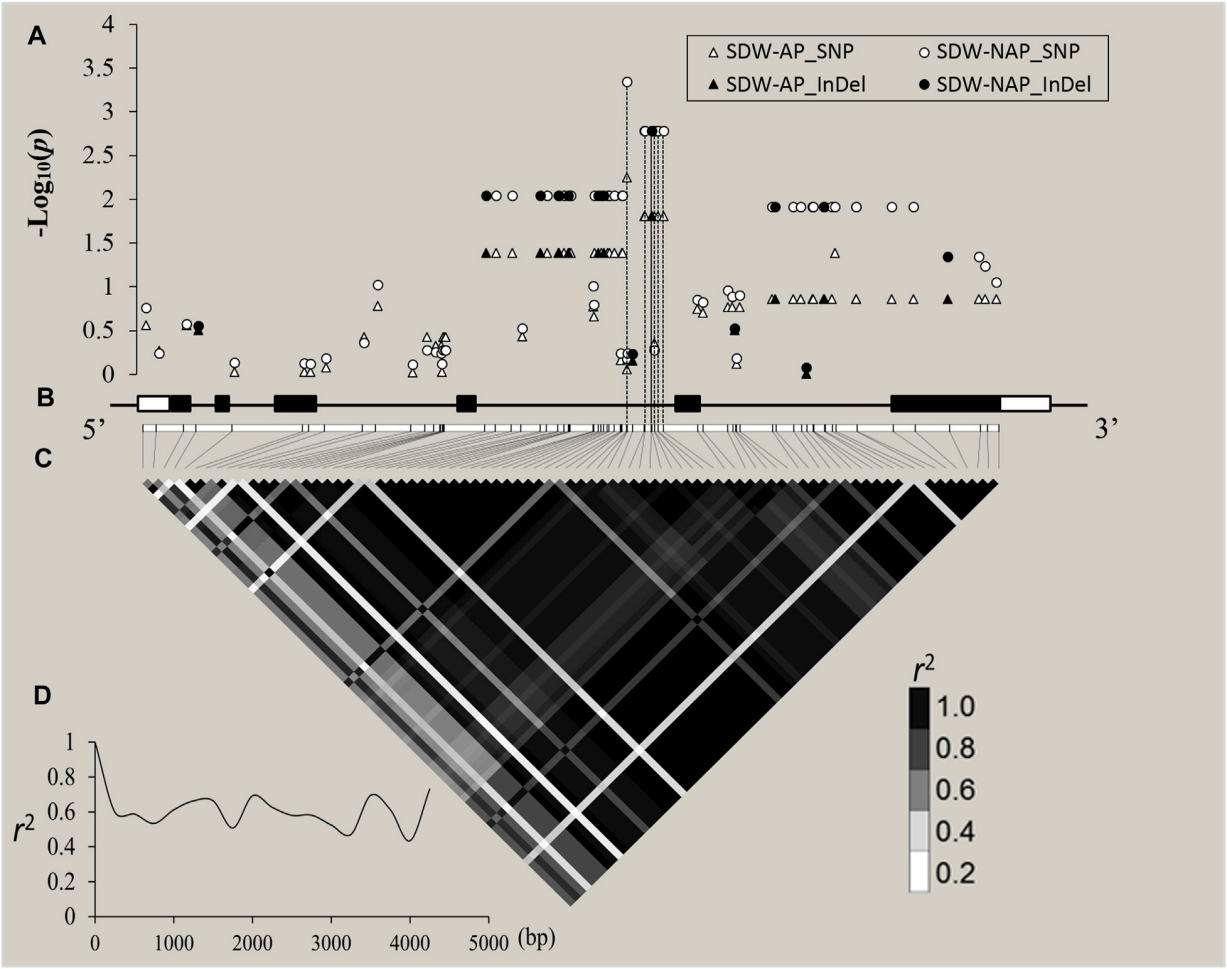
*ZmZFP126*-based association mapping and linkage disequilibrium (LD) analysis in 109 diverse maize inbreds. **(A)**: The white and black shapes (triangle and circle) indicated SNPs and Indel site, respectively. The *p* value is shown on a −log10 scale, and *p* ≥ 2 is considered a significant site. **(B)**: A schematic diagram of the entire gene structure is presented as the x-axis, including white and black boxes showing as UTRs and exons, respectively. C: The pattern of pairwise LD of DNA polymorphisms in the *ZmZFP126* gene. The color reflects the level of LD (*r* ^*2*^), and all polymorphic sites (MAF ≥0.05) are used. D: Decay speed of LD in the *ZmZFP126* gene. The x-axis indicated a full-length gene.

Through cloning and sequencing, *ZmZFP126* gene sequences of 109 inbred maize lines (Supplementary Table S1) from different countries and regions were obtained, and the gene sequences were analyzed. The SNPs and Indels loci in the sequence were extracted and analyzed for correlation with 20 agronomic traits of maize adult-plant stage (Supplementary Table S10; Supplementary Figure S2) and 19 shoot and root traits in the maize seedling stage under AP and NAP (Figure 5; Table 3). The results showed that 31 SNPs and 11 Indel loci of the *ZmZFP126* gene were significantly correlated with six agronomic traits (Supplementary Table S10). A total of 29 SNPs and 10 Indel loci of the gene were significantly correlated with an ear row number (ERN) (Supplementary Table S10; Supplementary Figure S2). In addition, from 24 SNPs and 6 Indel loci which were simultaneously significantly correlated with SDW-NAP, 8 SNPs and only one Indel were significantly correlated with a total dry weight under NAP (TDW-NAP) (Figure 5; Table 3). This result is consistent with the conclusion drawn from the correlation analysis, indicating that the *ZmZFP126* gene plays a key role in the development of the ear, the female flower, in corn, and low phosphorus stress. Linkage disequilibrium (LD) analysis shows that the LD degree of the *ZmZFP126* gene is very high from the 5′-end to the 3′-end. Almost the entire gene region forms an LD block, and the LD attenuation rate of the gene remains almost unchanged. It is at a high level of *r* ^*2*^ = 0.6 (Figure 5D). LD analysis results show that this gene is very conservative during evolution, and large fragment recombination rarely occurs.

**TABLE 3:** Amplified primers of *ZmZFP126* gene.

Primer sequence (5'-3′)	
Primer 1	
Forword	TGC​CGT​TGG​ATT​GCT​TTG​AG
Reverse	TGC​TAG​ACA​GGG​ATG​AGG​TGA
Primer 2	
Forword	TTG​TCT​GTC​CAC​ATC​CAG​GC
Reverse	AGC​ACA​ATA​GCA​GCA​TAC​CTT
Primer 3	
Forword	AAC​CAT​CAC​CAC​ACC​CCT​TG
Reverse	AAG​CAC​AAT​AGC​AGC​ATA​CCT
Primer 4	
Forword	TAT​GCC​CTT​TCC​CAG​CAT​GT
Reverse	ACA​CCG​AGT​GAT​TTG​CCA​GG

## Discussion

According to previous studies, the C2H2-ZF genes play an important regulatory role in various organisms (Takatsuji, 1999; Dinneny et al., 2006; Gamsjaeger et al., 2007). Several studies indicated the essential roles of C2H2-ZF genes in plants which involved in important biological processes (Xu et al., 2018; Gao et al., 2019; Han et al., 2020; Liu et al., 2020), while the C2H2-ZF gene family members functional analysis still limited in maize. Therefore, the main biological function of maize C2H2-ZFPs requires further exploration.

Gene duplication, including whole-genome replication, tandem replication, fragment replication, and gene transposition, is one of the main factors driving the evolution of genomes, generation of new functional genes, and the emergence of new species (Freeling, 2009). Genome-wide replication and tandem replication have important implications in the evolution of genomes and biological species (Fang et al., 2012). Wei et al. (2016) indicated that the maize C2H2-ZF gene family was probably extended by tandem duplication and segmental duplication. Tandem replication is prone to occur in hotspot regions of chromosome recombination, forming a cluster of genes that are similar in sequence and function, arranged in tandem with regard to head and tail (El-Sappah et al., 2021b). Previous studies have shown that tandem replication is closely related to the amplification of genes involved in biotic and abiotic stresses (Shiu et al., 2004; Maere et al., 2005; Rizzon et al., 2006). Freeling (2009) indicated that tandem replication tends to amplify genes at the top or end of the metabolic pathway along with dose-insensitive genes.

In mammals, the C2H2-ZF genes generally encode more ZF structures (Luchi, 2001), indicating a possible evolutionary process involving an increasing number of ZF structures in C2H2-ZFPs. Each of these two types of genes forms an independent group, indicating that they have evolved independently, and each type may have its independent functional characteristics. Previous studies have suggested that a ZF domain containing two Q-type ZF genes may become a gene containing four ZFs through replication or a single ZF gene by losing one domain (Kubo et al., 1998). The QA-type ZF domain continued evolving from the single ZF gene into a unique branch. Englbrecht et al. (2004) reached a similar conclusion through a comprehensive analysis of *Arabidopsis* C2H2-ZF genes. Their data also indicate that the Z-type ZF has recently evolved in the form of plant-specific ZFs.

The *ZmZFP056* gene was highly expressed at 12–24 DAP in whole seed and endosperm (Sekhon et al., 2011) (Supplementary Figure S1). These results indicated that the gene might participate in the biochemical process of seed starch accumulation in maize. Taking the results of the association analysis together, the chr2.S_197936812 SNP locus of this gene is significantly associated with kernel width (Supplementary Table S7). This gene is homologous to the *Arabidopsis AtIDD1/ENHYDROUS* gene (Figure 4). Previous functional analyses of the *AtIDD1* gene found that this gene promotes the transition to germination by regulating light and hormonal signaling during seed maturation (Feurtado et al., 2011; Fukazawa et al., 2014). In addition, the expression of the *ZmZFP056* gene under low-nitrogen conditions is significantly lower than in control conditions (Figure 2). Under drought conditions, the expression of this gene in the P1 inbred line (drought-tolerant) was significantly upregulated, but there was no significant change in the expression in P2 inbred lines (drought-sensitive). The association analysis results also showed that 18 SNP loci of this gene are significantly associated with maize seedling root and aboveground traits under NAP. The *ZmZFP033* gene was highly expressed in most other tissues or organs, especially at 12–24 DAP in the whole seed and endosperm. The association analysis results also showed that the *ZmZFP033* gene has seven SNP loci associated strongly with kernel width, and the chr2.S_17095383 locus is simultaneously significantly associated with 100-grain weight and ear diameter (Supplementary Table S7). This gene is also homologous to the *AtIDD1* gene (Figure 4). The expression patterns of *ZmZFP191*, *ZmZFP208*, *ZmZFP119*, and *ZmZFP153* are similar, being expressed at high levels at 12–24 DAP in whole seed and endosperm and in the leaves at different stages. Considering the association analysis results, three SNP loci in the *ZmZFP191* gene (chr9.S_149304614, chr9.S_149304735, and chr9.S_149304990) are all significantly associated with 100-grain weight. The chr9.S_149304614 locus is also significantly associated with ear leaf length, and the chr9.S_149304735 locus is significantly associated with kernel thickness and kernel width. The *ZmZFP208* gene has seven SNP loci (chr10.S_137499765, chr10.S_137500092, chr10.S_137500856, chr10.S_137500939, chr10.S_137500940, chr10.S_137501114, and chr10.S_137504374) that are significantly associated with 100-grain weight, of which chr10.S_137500092, chr10.S_137500856, and chr10.S_137501114 loci are significantly associated with ear row number, kernel thickness, and kernel width. The chr10.S_137504374 locus is also significantly associated with kernel thickness and kernel width. In addition, three SNP loci (chr10.S_137500293, chr10.S_137501104, and chr10.S_137502808) are significantly associated with ear row number, of which chr10.S_137500293 is also significantly associated with ear diameter. The chr10.S_137501104 and chr10.S_137502808 loci are also significantly associated with kernel length. The chr10.S_137505023 and chr10.S_137505138 loci are significantly associated with ear leaf length. The chr10.S_137500117 and PZE-110086343 loci are significantly associated with leaf number above the ear and kernel length, respectively. Four SNP loci (chr10.S_137499734, chr10.S_137499765, chr10.S_137500037, and chr10.S_137500215) are significantly associated with ear leaf width. Thirteen SNP loci in the *ZmZFP119* gene are significantly associated with kernel length, of which 11 are also significantly associated with ear leaf length. The chr7.S_146240353, chr7.S_146244619, and chr7.S_146244834 loci of the *ZmZFP153* gene are significantly associated with an ear row number, kernel width, and kernel thickness, respectively, and the chr7.S_146240306 locus is also significantly associated with kernel thickness and leaf number above the ear. Notably, the *ZmZFP208*, *ZmZFP119*, and *ZmZFP153* genes are homologous to the *AtIDD1* gene (Figure 4). The *ZmZFP144* gene was highly expressed at 16–24 DAP in embryos and immature cobs. This gene has nine SNP loci significantly associated with 100-grain weight and four SNP loci (chr7.S_117235871, chr7.S_117235888, chr7. S_117241506, and chr7.S_117241624) that are also significantly associated with kernel width. The *ZmZFP247* gene was highly expressed in meiotic tassels (V18_Meiotic tassel). The chr9.S_140945896 locus of this gene is also significantly associated with tassel main axis length, 100-grain weight, cob diameter, ear diameter, and ear leaf width. These results indicate that the expression of some genes is consistent with the results of the association analysis.

Under drought conditions, the *ZmZFP245*, *ZmZFP240*, and *ZmZFP202* genes are significantly downregulated in P1 and P2 inbred lines. The association analysis results showed that 12 SNP loci in the *ZmZFP245* gene are all significantly associated with R/S-AP. The chr7.S_8265307 locus of the *ZmZFP240* gene is significantly associated with RF-NAP and TRSA-NAP under NAP. The chr10.S_91956430 locus of the *ZmZFP202* gene is significantly associated with multiple seedling traits under NAP (e.g., RL2-NAP, RSA2-NAP, RV2-NAP, SDW-NAP, TDW-NAP, TFW-NAP, and TRV-NAP). The chr10.S_91956540 locus is significantly correlated with TFW-NAP, and the two SNP loci PZE-110049095 and chr10.S_91958988 are significantly correlated with RF-NAP. These results indicated the key role of the *ZmZFP245*, *ZmZFP240*, and *ZmZFP202* genes with various abiotic stresses.

The correlation analysis of maize C2H2-ZF genes showed that in most cases, the seed and ear traits are inherited together. In particular, the SNPs of *ZmZFP095* and *ZmZFP110* genes are only significantly correlated with the seed and ear traits. These two genes are thus highly likely to be involved in-ear and seed development, which directly affects plant yield. The seed traits, especially 100-grain weight, are an important indicator of corn yield. The kernel length, width, and thickness are directly related to 100-grain weight. The seed traits, in turn, are closely related to the ear traits, especially the ear row number, kernel number per row, and cob traits. Therefore, the seed and ear traits are generally inherited together. In addition, genes significantly associated with flowering traits were also significantly associated with traits related to plant height or ear height to some extent, suggesting that these genes may be involved in photoperiod responses.

Numerous researchers have used the tasseling stage, silking stage, anthesis stage, plant height, and ear height as important indicators to evaluate the photoperiod sensitivity of maize (Goodman, 1985; Ellis et al., 1992; Zhang and MUGO, 2001). In the present study, we found that 65% (19) of genes significantly associated with tassel traits are also significantly associated with seed traits, indicating a correlation between the two. The maize tassel grows at the top of the plant, which affords it substantial apical dominance. The tassel also develops earlier than the ears; hence, it has an advantage over the ears with regard to nutrient supply, as the tassel and the ears compete for nutrition (Yue et al., 2010; Ji et al., 2011). Researchers have also reported a relationship between corn tassel size and seed yield. It is generally considered that larger corn tassels need to consume more nutrients and can easily cause field canopy closure, which reduces ventilation and light transmission, thus affecting the improvement of grain yield. For example, Geraldi et al. (1985) have shown that the tassel branch number negatively correlates with yield. Duvick and Cassman (1999) focused on the changes that occurred in the post-Green Revolution era in the late 1960s, using the regression equations to estimate the magnitude of change in each maize hybrid trait from 1967 to 1991 and found that the tassel dry weight was reduced by 36%. The result indicates that the size of maize tassel is negatively correlated with yield. Field observations performed by Gao et al. (2007) on the Pioneer hybrid, which has been promoted in China in recent years, confirmed the significant reduction in tassel branch number.

In addition, we also found that 57% (28) of genes significantly associated with ear leaf traits were also significantly associated with the seed traits, indicating that the ear leaf traits are also directly related to grain yield. Previous studies have shown that the middle leaves of maize are crucial to the formation of grain yield. Studies have also shown that the three ear leaves (the ear leaf and the leaves from nodes above and below the fruiting ears) serve as effective photosynthetic layers of maize, which significantly accumulates dry matter (Zhao, 1981; Wei et al., 2000; Cui et al., 2009). Moreover, Bai et al. (1999) concluded that the ear leaf is the most important among the three ear leaves in influencing the grain yield.

We further revealed that 104 genes are significantly associated with agronomic traits in the adult plant stage, whereas 60 genes are significantly associated with traits in the seedling stage under normal and low P conditions, with 51 crossover genes between the two. These results indicated that C2H2-ZF genes are mainly involved in the reproduction and development of maize in the adult plant stage, whereas some are involved in response to both low P stress along with reproduction and development. Furthermore, these results also demonstrated the diversity of gene functions in the C2H2-ZF family genes, which implies that each gene may have multiple functions. Therefore, when studying the functions of C2H2-ZFPs, attention should not only be paid to their role in stress but also their important role in species reproduction and development. In addition, the C2H2-ZF genes that were significantly associated with flowering traits are rarely associated with traits under low P conditions; that is, ZFPs involved in photoperiod responses are rarely involved in low P stress. This suggests that stress and photoperiod response do not belong to the same regulatory system but rather represent independent physiological and biochemical systems. This result can guide future researchers in studying the functions of C2H2-ZFPs.

Finally, the *ZmZFP126* gene was identified as a poly-zinc finger structure gene located near the centromeric region of maize chromosome 6. This gene has two transcripts in the B73 genome. Transcript 1 encodes five C2H2-ZFs, whereas transcript 2 encodes four. The correlation analysis revealed that this gene is significantly associated with the SDW-NAP and TDW-NAP. Notably, this gene has no significant correlation with RDW-NAP, which suggests that under NAP, this C2H2-ZF gene can also regulate the growth of the aboveground parts of inbred maize lines that do not have highly developed roots. Moreover, the LD level of this gene is very high throughout the entire gene region, indicating that it is evolutionarily conserved and is rarely affected by genomic recombination. This characteristic of the *ZmZFP126* gene may be related to its location on the chromosome, specifically, near the centromere on maize chromosome 6, as the centromere region is considered to have a relatively low recombination rate.

## Conclusion

The C2H2-zinc finger proteins (ZFP) comprise a large family of transcription factors with various functions in biological processes. C2H2-ZF genes’ function in maize is still limited. Thus, we performed an evolution and functional prediction analysis of the maize C2H2-ZF genes. Moreover, the association analysis and the relationship between maize C2H2-ZF genes and other C2H2-ZF genes with known functions showed the essential role of maize genes, especially *ZmZFP126*. Thus, we select this gene for cloning and the discovery of favorable allelic variation. This characteristic of the *ZmZFP126* gene may be related to its location on the chromosome, specifically, near the centromere on maize chromosome 6, as the centromere region is considered to have a relatively low recombination rate. Finally, our study about the C2H2-ZF family genes in the maize genome will contribute to a comprehensive understanding of the evolution of the C2H2-ZF domain and the roles of these genes during the maize life cycle and growth.

## Data Availability

The datasets presented in this study can be found in online repositories. The names of the repository/repositories and accession number(s) can be found below: European Nucleotide Archive, accession no: PRJEB48267. Our study unique name is ena-STUDY-Yibin University-22-10-2021-07:58:56:029-331.

## Supplementary Material

Supplementary Figure 1 Expression profiles of C2H2-ZF genes in eleven tissue or organs across maize different developmental stages. The red, green, and black represent high, low, and medium gene expression, respectively.

Supplementary Figure 2
*ZmZFP126*-based association mapping and LD analysis in 109 diverse maize inbreds. **(A)**: Circle and triangle indicate SNPs and Indel site, respectively, and red circle and triangle represent significant sites. The P-value is shown on a −log10 scale. **(B)**: A schematic diagram of the entire gene structure is presented as the x-axis, including white and black boxes showing as UTRs and exons, respectively. **(C)**: The pattern of pairwise LD of DNA polymorphisms in the *ZmZFP126* gene. The color reflects the level of LD (*r* ^*2*^), and all polymorphic sites (MAF ≥ 0.05) are used. **(D)**: Decay speed of LD in the *ZmZFP126* gene. The x-axis indicated a full-length gene.
