# Supplementary figures and images for: In Silico Functional Prediction and Expression Analysis of C2H2 Zinc-Finger Family Transcription Factor Revealed Regulatory Role of ZmZFP126 in Maize Growth
Source: Front Genet. 2021 Nov 5;12:770427. doi: 10.3389/fgene.2021.770427 (PMC8602080; doi:10.3389/fgene.2021.770427)

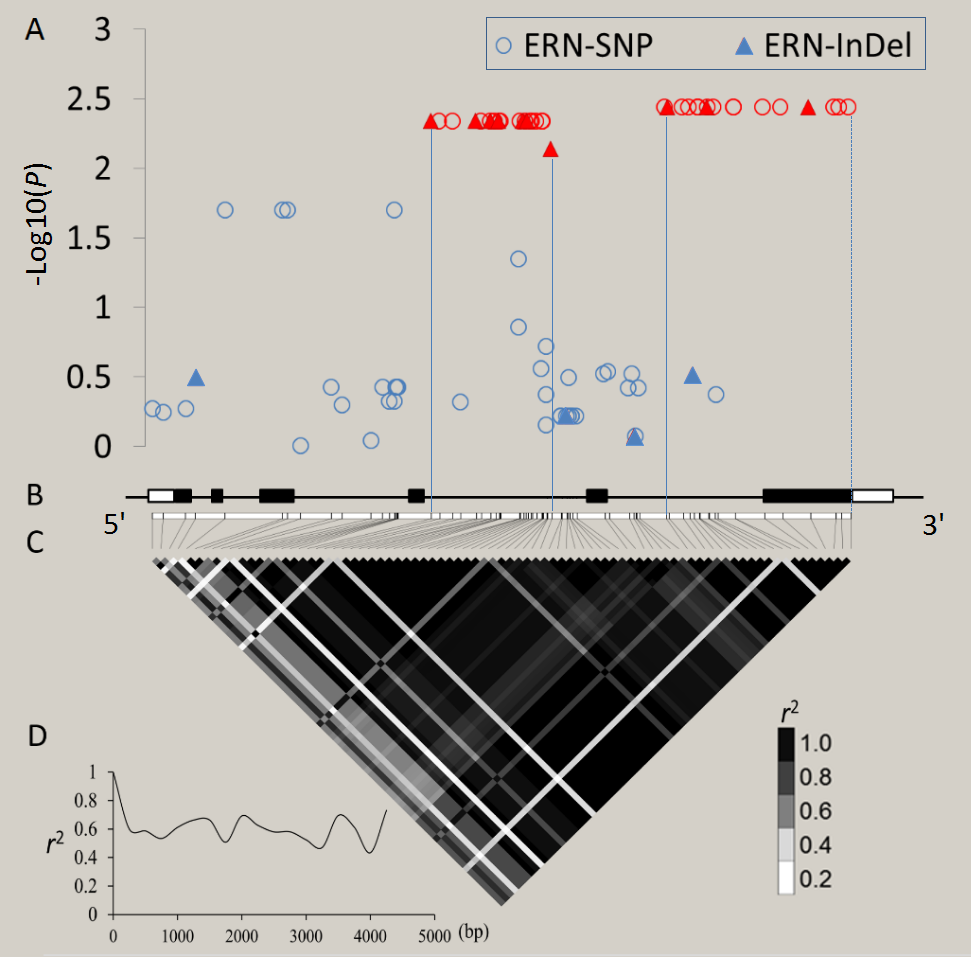

Supplement: Supplementary file 5 [file Image2.TIF]
