# Supplementary material for: In Silico Functional Prediction and Expression Analysis of C2H2 Zinc-Finger Family Transcription Factor Revealed Regulatory Role of ZmZFP126 in Maize Growth
Source: Front Genet. 2021 Nov 5;12:770427. doi: 10.3389/fgene.2021.770427 (PMC8602080; doi:10.3389/fgene.2021.770427)
Supplement: Supplementary file 8 [file Table9.DOCX]

**Supplementary Table 9** Sixty-seven C2H2-ZF genes with known functions and their homologous genes in maize

| **Number** | **Gene name** | **Gene ID** | **Function description** | **Species** | **Maize Ortholog** |
| --- | --- | --- | --- | --- | --- |
| 1 | *AtZFP1([Joseph et al., 2014](#_ENREF_37))* | AT1G80730 | Downstream of GL2 to repress root hair initiation and elongation by directly suppressing bHLH genes | *Arabidopsis thaliana* | *ZmZFP015*  *ZmZFP025*  *ZmZFP107*  *ZmZFP114*  *ZmZFP162* |
| 2 | *AtSIZ1([Han et al., 2019](#_ENREF_27); [Kwak et al., 2019](#_ENREF_52); [Kim et al., 2016a](#_ENREF_42); [Kim et al., 2016b](#_ENREF_45); [Kim et al., 2015b](#_ENREF_46); [Jin et al., 2008](#_ENREF_36); [Miura et al., 2005](#_ENREF_73); [Park et al., 2011](#_ENREF_79))* | AT3G25910 | AtSIZ1 improves salt tolerance by maintaining ionic homeostasis and osmotic balance in *Arabidopsis*; E3 SUMO ligase AtSIZ1 regulates the cruciferin content of *Arabidopsis* seeds; COP1 controls abiotic stress responses by modulating AtSIZ1 function through Its E3 ubiquitin ligase activity; The E3 SUMO ligase AtSIZ1 functions in seed germination in *Arabidopsis*; E3 SUMO ligase AtSIZ1 positively regulates SLY1-mediated GA signalling and plant development; AtSIZ1 regulates flowering by controlling a salicylic acid-mediated floral promotion pathway and through affects on FLC chromatin structure; AtSIZ1 controls phosphate deficiency responses; *Arabidopsis* nitrate reductase activity is stimulated by the E3 SUMO ligase AtSIZ1. | *Arabidopsis thaliana* | *ZmZFP225.1*  *ZmZFP233*  *ZmZFP235* |
| 3 | *AtCZS([Krichevsky et al., 2007](#_ENREF_50); [Grewal et al., 2012](#_ENREF_25))* | AT2G23740 | AtSWP1 and AtCZS, interact with each other in plant cells and repress expression of a negative regulator of flowering, FLOWERING LOCUS C (FLC) via an autonomous, vernalization-independent pathway. | *Arabidopsis thaliana* | *ZmZFP096*  *ZmZFP085*  *ZmZFP120* |
| 4 | *AtZFP3([Joseph et al., 2014](#_ENREF_37))* | AT5G25160 | Interferes with abscisic acid and light signaling in seed germination and plant development; Involved in salt stress and osmotic stress response | *Arabidopsis thaliana* | *ZmZFP006.1* |
| 5 | *AtZFP5([An et al., 2012b](#_ENREF_2); [Zhou et al., 2011](#_ENREF_120); [Zhou et al., 2012](#_ENREF_119))* | AT1G10480 | associates with ethylene signaling to regulate the phosphate and potassium deficiency-induced root hair development in *Arabidopsis*. | *Arabidopsis thaliana* | *ZmZFP054* |
| 6 | *AtZFP6([Zhou et al., 2013](#_ENREF_121); [Joseph et al., 2014](#_ENREF_37))* | AT1G67030 | regulates trichome initiation by integrating gibberellin and cytokinin signaling in *Arabidopsis* thaliana | *Arabidopsis thaliana* | *ZmZFP071* |
| 7 | *AtZFP8([Gan et al., 2007](#_ENREF_22))* | AT2G41940 | *AtZFP8* gene control trichome cell development through GA and cytokinin signalling in *Arabidopsis* | *Arabidopsis thaliana* | *ZmZFP054*  *ZmZFP122.1*  *ZmZFP089*  *ZmZFP098*  *ZmZFP149* |
| 8 | *AtZFP10([Dinkins et al., 2002](#_ENREF_14))* | AT2G37740 | Tobacco plants overexpressing the *AtZFP10* gene displayed dwarfing, abnormal leaf phenotypes and early flowering that correlated with the level of expression of the *AtZFP10* gene | *Arabidopsis thaliana* | *ZmZFP032*  *ZmZFP087*  *ZmZFP112*  *ZmZFP123*  *ZmZFP135*  *ZmZFP203*  *ZmZFP207*  *ZmZFP141* |
| 9 | *AtZFP11([Dinkins et al., 2003](#_ENREF_15))* | AT2G42410 | The *AtZFP11* gene was found to be expressed in flowers, axillary meristems, roots and stems of *Arabidopsis* by RT-PCR, Ectopic expression of the *AtZFP11* gene in tobacco resulted in abnormal tobacco plants that were dwarfed, had abnormal leaf morphology, flowered early and most of the plants were sterile. | *Arabidopsis thaliana* | *ZmZFP032* |
| 10 | *ZAT1([Song et al., 2020](#_ENREF_92))* | AT1G02030 | Overexpression of ZAT1 induced the ectopic expression of PUTATIVE ASPARTIC PROTEASE3 involved in the programmed cell death. The EAR motif was essential for the growth inhibition by ZAT1. The growth inhibition by ZAT1 was shared by ZAT4 and ZAT9, the ZAT1 homologues. | *Arabidopsis thaliana* | *ZmZFP179*  *ZmZFP034* |
| 11 | *ZAT4([Song et al., 2020](#_ENREF_92))* | AT2G45120 | The growth inhibition by ZAT1 was shared by ZAT4 and ZAT9, the ZAT1 homologues. | *Arabidopsis thaliana* | *ZmZFP179*  *ZmZFP034* |
| 12 | *ZAT6([Devaiah et al., 2007](#_ENREF_13); [Chen et al., 2016](#_ENREF_7); [Shi et al., 2018a](#_ENREF_90); [Liu et al., 2013](#_ENREF_63))* | AT5G04340 | ZAT6 regulate root development and nutrient stress responses; Phosphorylation of the zinc finger transcriptional regulator ZAT6 by MPK6 regulates *Arabidopsis* seed germination under salt and osmotic stress; ZAT6 positively regulates Cadmium tolerance through the glutathione-dependent pathway in *Arabidopsis*; ZAT6 is essential for hydrogen peroxide induction of anthocyanin synthesis in *Arabidopsis*; AtZAT6 plays important roles in plant development and positively modulates biotic and abiotic stress resistance by activating the expression levels of salicylic acid-related genes and CBF genes | *Arabidopsis thaliana* | *ZmZFP013*  *ZmZFP010*  *ZmZFP012*  *ZmZFP157*  *ZmZFP185*  *ZmZFP186*  *ZmZFP011* |
| 13 | *ZAT9([Song et al., 2020](#_ENREF_92))* | AT3G60580 | The growth inhibition by ZAT1 was shared by ZAT4 and ZAT9, the ZAT1 homologues. | *Arabidopsis thaliana* | *ZmZFP034* |
| 14 | *STZ/ZAT10([Nguyen et al., 2011](#_ENREF_76); [Mittler et al., 2006](#_ENREF_72))* | AT1G27730 | ZAT10 in *Arabidopsis* was found to elevate the expression of reactive oxygen-defense transcripts and to enhance the tolerance of plants to salinity, heat and osmotic stress. Surprisingly, knockout and RNAi mutants of ZAT10 were also more tolerant to osmotic and salinity stress. | *Arabidopsis thaliana* | *ZmZFP105*  *ZmZFP012*  *ZmZFP186*  *ZmZFP026*  *ZmZFP011*  *ZmZFP010* |
| 15 | *ZAT11([Liu et al., 2014](#_ENREF_62); [Gechev et al., 2005](#_ENREF_24))* | AT2G37430 | ZAT11 negative regulator of nickel ion tolerance in *Arabidopsis* | *Arabidopsis thaliana* | *ZmZFP008* |
| 16 | *ZAT18([Yin et al., 2017](#_ENREF_112))* | AT3G53600 | Transcription factor involved in stress responses (Probable); Positive regulator of the jasmonic acid (JA)- mediated signaling pathway; Triggers the up-regulation of LOX3, VSP2, PAL1 and PAL2 in a JA-dependent manner; Promotes drought and osmotic stress tolerance by preventing reactive oxygen species (ROS) production (e.g. H2O2). | *Arabidopsis thaliana* | *ZmZFP083*  *ZmZFP084*  *ZmZFP176* |
| 17 | *AtGIS([Gan et al., 2006](#_ENREF_21); [Gan et al., 2007](#_ENREF_22); [An et al., 2012a](#_ENREF_1); [Sun et al., 2013](#_ENREF_93))* | AT3G58070 | Probable transcription factor required for the initiation of inflorescence trichomes in response to gibberellin (GA); Mediates the induction of GL1 expression by GA in inflorescence organs and is antagonized in its action by the DELLA repressor GAI. Acts upstream of the trichome initiation regulators GL1 and GL3, and downstream of the GA signaling repressor SPINDLY (SPY); Does not play a significant role in the cytokinin response; Controls trichome branching through GA signaling; Acts downstream of the key regulator STICHEL (STI) in an endoreduplication-independent pathway; Controls trichome cell division indirectly by acting downstream of a key endoreduplication regulator SIAMESE (SIM). | *Arabidopsis thaliana* | *ZmZFP098*  *ZmZFP130* |
| 18 | *AtGIS2([Gan et al., 2007](#_ENREF_22))* | AT5G06650 | Probable transcription factor required for the initiation of inflorescence trichomes in response to gibberellin and cytokinin. Is not involved in the regulation of trichome branching. Is functionally equivalent to ZFP8. | *Arabidopsis thaliana* | *ZmZFP122.1* |
| 19 | *AtGIS3([Sun et al., 2015](#_ENREF_94))* | AT1G68360 | Probable transcription factor required for the initiation of inflorescence trichomes in response to gibberellin and cytokinin. Acts upstream of GIS, GIS2, ZFP8, and the trichome initiation factors GL1 and GL3. Binds the promoter region of GIS and GIS2, which may be direct targets of GIS3. Plants over-expressing GIS3 have increased trichome densities in sepals, cauline leaves, lateral branches, main inflorescence stems, and have ectopic trichomes on carpels. | *Arabidopsis thaliana* | *ZmZFP071*  *ZmZFP160* |
| 20 | *GsGIS3([Liu et al., 2020](#_ENREF_64))* | XP_003536676 | Improves aluminum tolerance in *Arabidopsis* | *Glycine soja* | *ZmZFP071*  *ZmZFP160* |
| 21 | *GsZFP1([Luo et al., 2012b](#_ENREF_69); [Luo et al., 2012a](#_ENREF_68); [Tang et al., 2013](#_ENREF_98); [Yu et al., 2014](#_ENREF_114))* | A0A445IUK6 | GsZFP1 is a positive regulator of plant tolerance to cold and drought stress; Overexpression of GsZFP1 enhances salt and drought tolerance in transgenic alfalfa (Medicago sativa L.); Over-expression of GsZFP1 reduces ABA sensitivity and decreases stomata size. | *Glycine soja* | *ZmZFP038* |
| 22 | *KNUCKLES*(KNU)([Payne et al., 2004](#_ENREF_80)) | AT5G14010 | May function as a transcriptional repressor of cellular proliferation that regulates floral determinacy and relative size of basal pattern elements along the proximo-distal axis of the developing gynoecium. | *Arabidopsis thaliana* | *ZmZFP136* |
| 23 | *ZjZFN1([Teng et al., 2018](#_ENREF_100))* | AMC13792.1 | *ZjZFN1* gene is improved salt tolerance in *Arabidopsis* | *Zoysia japonica* | *ZmZFP026*  *ZmZFP105* |
| 24 | *AtYY1([Li et al., 2016b](#_ENREF_59))* | AT4G06634 | *AtYY1* gene is a novel negative regulator of the *Arabidopsis* ABA response network | *Arabidopsis thaliana* | *ZmZFP126*  *ZmZFP093*  *ZmZFP117* |
| 25 | RABBIT EARS (RBE)([Takeda et al., 2004](#_ENREF_97)) | AT5G06070 | Probable transcriptional regulator essential for petal development. Required for the early development of the organ primordia of the second whorl. Acts downstream of AP1 and PTL. | *Arabidopsis thaliana* | *ZmZFP135* |
| 26 | *SlZF3([Li et al., 2018](#_ENREF_60))* | Solyc06g075780 | SlZF3 regulates AsA synthesis and salt tolerance by interacting with CSN5B | *Solanum lycopersicum* | ZmZFP079 |
| 27 | *GhSTOP1([Kundu et al., 2018](#_ENREF_51))* | LOC107899819 | essential for aluminum and proton stress tolerance and lateral root initiation in cotton | Gossypium hirsutum L. | ZmZFP031  ZmZFP118.1  ZmZFP194.1  ZmZFP077  ZmZFP171 |
| 28 | *AtSTOP1([Iuchi et al., 2007](#_ENREF_34); [Sawaki et al., 2009](#_ENREF_89))* | AT1G34370 | *STOP1* plays a critical role in *Arabidopsis* tolerance to major stress factors in acid soils; *STOP1* is critical for proton tolerance in *Arabidopsis* and coregulates a key gene in aluminum tolerance | *Arabidopsis thaliana* | *ZmZFP031*  *ZmZFP118.1*  *ZmZFP194.1*  *ZmZFP077*  *ZmZFP171* |
| 29 | *SbSTOP1([Huang et al., 2018](#_ENREF_31); [Gao et al., 2019](#_ENREF_23))* (SbSTOP1a, SbSTOP1b, SbSTOP1c, SbSTOP1d) | Sb01g001950.1, Sb04g023670.1, Sb07g023890.1, Sb03g041170.1 | *STOP1* plays an important role in Al tolerance in sweet sorghum | *Sorghum bicolor* | *ZmZFP031*  *ZmZFP118.1*  *ZmZFP194.1*  *ZmZFP077*  *ZmZFP171* |
| 30 | *GmZFP3([Zhang et al., 2016](#_ENREF_118))* | Glyma07g02880 | Negatively regulates drought responses in transgenic *Arabidopsis* | *Glycine max* | *ZmZFP006.1* |
| 31 | *GmZAT4([Sun et al., 2019](#_ENREF_96))* | A0A059T9X4 | GmZAT4 enhances PEG and NaCl stress tolerances in *Arabidopsis thaliana* | *Glycine max* | *ZmZFP184* |
| 32 | *GmZF1([Yu et al., 2014](#_ENREF_114))* | Q4FH86 | GmZF1 enhanced cold tolerance in transgenic *Arabidopsis* | *Glycine max* | *ZmZFP008*  *ZmZFP187*  *ZmZFP125* |
| 33 | *MAZ1([Lyu et al., 2019](#_ENREF_70))* | AT5G15480 | MAZ1 is essential for intine formation and exine pattern. | *Arabidopsis thaliana* | *ZmZFP062* |
| 34 | *IDD15/SGR5([Morita et al., 2006](#_ENREF_74); [Kim et al., 2015a](#_ENREF_43); [Tanimoto et al., 2008](#_ENREF_99); [Cui et al., 2013](#_ENREF_11))* | AT2G01940 | Transcription factor involved in inflorescence stems gravitropism, probably by regulating starch accumulation in amyloplasts of graviperceptive cells. Required for stem circumnutation movements. Regulates lateral organ morphogenesis and gravitropic responses. Acts cooperatively with IDD16 to control silique and branche orientation. Involved in the establishment of auxin gradients through the regulation of auxin biosynthesis and transport. | *Arabidopsis thaliana* | *ZmZFP005*  *ZmZFP189*  *ZmZFP021*  *ZmZFP055*  *ZmZFP150* |
| 35 | *IDD1/ENY([Feurtado et al., 2011](#_ENREF_19); [Fukazawa et al., 2014](#_ENREF_20); [Yamauchi et al., 1997](#_ENREF_110); [Tanimoto et al., 2008](#_ENREF_99))* | AT5G66730 | Transcription factor promoting the transition to germination by regulating light and hormonal signaling during seed maturation. Acts as a positive regulator of phytochrome and/or gibberellin action. | *Arabidopsis thaliana* | *ZmZFP003*  *ZmZFP068*  *ZmZFP168*  *ZmZFP033.1*  *ZmZFP161*  *ZmZFP086.1*  *ZmZFP023.1*  *ZmZFP153*  *ZmZFP208.1*  *ZmZFP119*  *ZmZFP069*  *ZmZFP116*  *ZmZFP155*  *ZmZFP060*  *ZmZFP075*  *ZmZFP056*  *ZmZFP024* |
| 36 | *SCOF-1([Kim et al., 2001b](#_ENREF_41); [Kim et al., 2001a](#_ENREF_40))* | AAB39638.1 | SCOF-1 enhances cold tolerance in transgenic plants; the elevation of the SCOF-1 transcript level by cold stress is associated with both transcriptional activation and post-transcriptional mRNA stability under a low temperature. | *Glycine max* | *ZmZFP105*  *ZmZFP186*  *ZmZFP026* |
| 37 | *StZFP1([Tian et al., 2010](#_ENREF_101); [Lawrence et al., 2014](#_ENREF_55))* | ABK78777.1 | *StZFP1* responds to biotic and abiotic stress, plays a role in salt tolerance | *Solanum tuberosum L.* | *ZmZFP105* |
| 38 | *StZFP2([Lawrence et al., 2014](#_ENREF_55); [Lawrence and Novak, 2018a](#_ENREF_53); [Lawrence and Novak, 2018b](#_ENREF_54))* | PGSC0003DMP400027271 | Expression studies demonstrate that *StZFP2* transcript is also induced by tobacco hornworm and Colorado potato beetle; Over-expression of *StZFP2* in *Solanum tuberosum* L. var. Kennebec (potato) inhibits growth of Tobacco Hornworm larvae (THW, *Manduca sexta* L.) | *Solanum tuberosum L.* | *ZmZFP079*  *ZmZFP008*  *ZmZFP172* |
| 39 | *JcZFP8([Shi et al., 2018b](#_ENREF_91))* | A0A067KE06 | Regulation of trichome development in tobacco by JcZFP8 | *Jatropha curcas* | *ZmZFP122.1*  *ZmZFP054*  *ZmZFP098*  *ZmZFP089*  *ZmZFP004*  *ZmZFP149*  *ZmZFP175*  *ZmZFP130* |
| 40 | *Hair/H([Chang et al., 2018](#_ENREF_6))* | Solyc10g078970.1.1 | Hair regulates multicellular trichome formation in tomato | *Solanum lycopersicum* | *ZmZFP122.1* |
| 41 | *SUF4([Resentini et al., 2017](#_ENREF_86); [Kim and Michaels, 2006](#_ENREF_47); [Kim et al., 2006](#_ENREF_44); [Choi et al., 2009](#_ENREF_8); [Choi et al., 2011](#_ENREF_9))* | AT1G30970 | SUF4 represses flowering by transcriptional activation of *Arabidopsis* FLOWERING LOCUS C; Sequence-specific DNA binding factor that recognizes the 5'-CCAAATTTTAAGTTT-3' sequence. Recruits the FRI-C complex to the FLC promoter. Required for FRI-mediated FLC activation, but has no effect on the expression of MAF1, MAF2, MAF3, MAF5, UFC and CO. Dispensable for the reactivation of FLC in early embryogenesis, but required to maintain high levels of FLC expression in later embryonic and vegetative development. | *Arabidopsis thaliana* | *ZmZFP058* |
| 42 | JAGGED (JAG)([Dinneny et al., 2004](#_ENREF_18); [Ohno et al., 2004](#_ENREF_78); [Dinneny et al., 2006](#_ENREF_17); [Xu et al., 2008](#_ENREF_106); [Dinneny et al., 2005](#_ENREF_16)) | AT1G68480 | Controls the morphogenesis of lateral organs. Functions in lateral organ shape and is sufficient to induce proliferation and growth of lateral organ tissue. Is necessary and sufficient for bract formation, but its expression is excluded from the cryptic bract, which could be a cause of bractless flowers in *Arabidopsis*. Participates with FIL and YAB3 in regulating valve margin development. Functions with JGL to define stamen and carpel shape. Functions with AS1 and AS2 in the sepal and petal primordia to repress boundary-specifying genes for normal development of the organs. | *Arabidopsis thaliana* | *ZmZFP159*  *ZmZFP071*  *ZmZFP160* |
| 43 | NUBBIN (NUB) /JGL([Dinneny et al., 2006](#_ENREF_17)) | AT1g13400 | Acts with JAG to promote growth and patterning in stamens and carpels. Promotes the growth of the abaxial and adaxial sides of floral organs. Promotes the growth of the pollen-bearing microsporangia in anthers, the carpel walls of the gynoecium and the establishment of the correct number of cell layers in carpel walls. Promotes leaf blade growth and trichome development. | *Arabidopsis thaliana* | *ZmZFP159* |
| 44 | *ARS1([Baek et al., 2015](#_ENREF_3))* | AT3G02860 | Essential for breaking seed dormancy before seed germination. Prevents reactive oxygen species (ROS) accumulation in response to abscisic acid (ABA) and oxidative stress, probably by repressing the accumulation of ABA-induced ROS-scavenging enzymes. | *Arabidopsis thaliana* | No |
| 45 | *UPRIGHT ROSETTE (URO)([Sun et al., 2010](#_ENREF_95); [Yue et al., 2000](#_ENREF_116))* | AT3G23140 | Overexpression of the *Arabidopsis* gene *UPRIGHT ROSETTE* reveals a homeostatic control for indole-3-acetic acid. | *Arabidopsis thaliana* | *ZmZFP137* |
| 46 | *SERRATE* (*SE*)([Prigge and Wagner, 2001](#_ENREF_81); [Yang et al., 2006](#_ENREF_111)) | AT2G27100 | Required for Normal Shoot Development; controls leaf development, meristem activity, inflorescence architecture and developmental phase transition; The RNA-binding proteins HYL1 and SE promote accurate in vitro processing of pri-miRNA by DCL1 | *Arabidopsis thaliana* | *ZmZFP214*  *ZmZFP227*  *ZmZFP238*  *ZmZFP234* |
| 47 | *BcMF20([Han et al., 2018](#_ENREF_28); [Han et al., 2011](#_ENREF_29))* | ADK92391.1 | BcMF20 may act as a part of regulation mechanisms of TAZ1 and MS1. Together they play a role in a genetic pathway in the tapetum to act on proliferation of tapetal cells and keep the normal development of pollens. | *Brassica campestris ssp.* chinensis | *ZmZFP034* |
| 48 | *WIP2/NTT([Chung et al., 2013](#_ENREF_10); [Marsch-Martínez et al., 2014](#_ENREF_71))* | AT3G57670 | In ntt mutants, pollen tubes grow more slowly and/or terminate prematurely, and lateral divergence is accentuated over apical-to-basal movement | *Arabidopsis thaliana* | *ZmZFP132*  *ZmZFP163*  *ZmZFP049*  *ZmZFP139*  *ZmZFP151*  *ZmZFP020*  *ZmZFP100*  *ZmZFP237*  *ZmZFP131*  *ZmZFP177* |
| 49 | *GAZ ([Lee et al., 2016](#_ENREF_56))* | AT2G18490 | GAZ was involved in the transcriptional regulation of ABA and GA homeostasis | *Arabidopsis thaliana* | *ZmZFP018* |
| 50 | *SUPERMAN/FLO10/SUP([Sakai et al., 1995](#_ENREF_88); [Yun et al., 2002](#_ENREF_117); [Isernia et al., 2003](#_ENREF_32); [Ito et al., 2003](#_ENREF_33); [Prunet et al., 2017](#_ENREF_82); [Xu et al., 2018](#_ENREF_108))* | AT3G23130 | Probable transcriptional regulator considered as cadastral protein that acts indirectly to prevent the B class homeotic proteins APETALA3 and perhaps PISTILLATA from acting in the gynoecial whorl. Principal function is to balance cell proliferation in the third and fourth whorls of developing flowers thereby maintaining the boundary between stamens and carpels. May fulfill this role by repressing genes implicated in cell division. Plays equally a role in the determinacy of the floral meristem. Is also required for normal ovule development. | *Arabidopsis thaliana* | *ZmZFP207*  *ZmZFP135*  *ZmZFP032*  *ZmZFP087* |
| 51 | *CsSUP([Wu et al., 2014](#_ENREF_104))* | Cucsa.255130.1 | Cucumber *SUPERMAN* has conserved function in stamen and fruit development and a distinct role in floral patterning. | Cucumis sativus L. | ZmZFP087  ZmZFP135  ZmZFP032  ZmZFP207  ZmZFP112 |
| 52 | *PhSUP1([Nakagawa et al., 2004](#_ENREF_75))* | BAD11142.1 | PhSUP1 transcripts occurred in the basal region of wild-type flowers around developing organ primordia in whorls 2 and 3 as well as in the funiculus of the ovule, concave regions of the placenta, and interthecal regions of developing anthers. Overexpression of PhSUP1 in *P. hybrida* resulted in size reduction of petals, leaves, and inflorescence stems. | *Petunia hybrida* | *ZmZFP135*  *ZmZFP087*  *ZmZFP032*  *ZmZFP207* |
| 53 | *SlSUP([Kazama et al., 2009](#_ENREF_39))* | BAH59432.1 | *SlSUP* gene is exclusively expressed in female flowers of the dioecious plant *Silene latifolia*. | *Silene latifolia* | *ZmZFP135*  *ZmZFP032*  *ZmZFP207*  *ZmZFP087*  *ZmZFP112* |
| 54 | *STAMENLESS 1* (*SL1*)([Xiao et al., 2009](#_ENREF_105)) | Os01g0129200, LOC_Os01g03840 | Regulates floral organ identity and cell proliferation in the inner floral whorls. Probably specifies the identities of lodicule and stamen through positive regulation of MADS16 expression. May contribute to morphogenesis by suppressing OSH1 expression in the lateral organs. | *Oryza sativa* | *ZmZFP159* |
| 55 | *DAZ1/DAZ2/ZAT2([Borg et al., 2014](#_ENREF_4))* | AT2G17180 | Mediates the regulation of male germ cell division by DUO1 | *Arabidopsis thaliana* | *ZmZFP099*  *ZmZFP019*  *ZmZFP179* |
| 56 | *SAZ([Jiang et al., 2007](#_ENREF_35))* | AT4G17810 | SAZ is involved in the negative regulation of ABA-mediated signaling | *Arabidopsis thaliana* | *ZmZFP135* |
| 57 | *FIS2([Guitton et al., 2004](#_ENREF_26); [Luo et al., 2000](#_ENREF_66); [Qiu et al., 2017](#_ENREF_83); [Jullien et al., 2006](#_ENREF_38))* | AT2G35670 | Polycomb group (PcG) protein. PcG proteins act by forming multiprotein complexes, which are required to maintain the transcriptionally repressive state of homeotic genes throughout development. PcG proteins are not required to initiate repression, but to maintain it during later stages of development. They probably act via the methylation of histones, rendering chromatin heritably changed in its expressibility. Required to prevent the proliferation of the central cell by repressing unknown target genes before fertilization. Regulates the anteroposterior organization of the endosperm. | *Arabidopsis thaliana* | *ZmZFP241.1* |
| 58 | *ZmMRPI-1* (*ZmZFP174*  )*([Royo et al., 2009](#_ENREF_87))* | GRMZM2G139160 | ZmMRP-1 is a single MYB-domain transcription factor specifically expressed in the transfer cell layer of the maize endosperm, where it directly regulates the expression of a number of transfer cell specific genes and very likely contributes to the regulation of the transfer cell differentiation process. ZmMRPI-1 and ZmMRPI-2 interact with ZmMRP-1 and modulate its activity on transfer cell specific promoters. | *Zea mays* | *ZmZFP080*  *ZmZFP231*  *ZmZFP210*  *ZmZFP044*  *ZmZFP101*  *ZmZFP202*  *ZmZFP042.2*  *ZmZFP007.1* |
| 59 | *ZmMRPI-2* (*ZmZFP080*)*([Royo et al., 2009](#_ENREF_87))* | GRMZM2G105224 | Same as ZmMRPI-1 | *Zea mays* | *ZmZFP174*  *ZmZFP231*  *ZmZFP210*  *ZmZFP044*  *ZmZFP101*  *ZmZFP202*  *ZmZFP042.2*  *ZmZFP007.1* |
| 60 | *LATE([Weingartner et al., 2011](#_ENREF_103))* | AT5G48890 | Acts as a transcriptional repressor. Prevents the photoperiodic and circadian clock-dependent transition to flowering in long days (LD) by repressing the expression of flowering time genes (e.g. FT, GI and CO) in the leaf vasculature, and by interfering with floral meristem identity genes at the apex (e.g. SOC1 and LFY). | *Arabidopsis thaliana* | *ZmZFP017*  *ZmZFP027*  *ZmZFP104*  *ZmZFP136* |
| 61 | *ART1/STAR3([Tsutsui et al., 2011](#_ENREF_102); [Yamaji et al., 2009](#_ENREF_109))* | Os12g0170400, LOC_Os12g07280 | Transcriptional activator that regulates the expression of genes involved in aluminum (Al) tolerance. Binds to the promoter of STAR1 and regulates transcript expression of STAR1 STAR2 and ART1 required for Al tolerance. | *Oryza sativa* | *ZmZFP194.1*  *ZmZFP171.1*  *ZmZFP077*  *ZmZFP118.1*  *ZmZFP031* |
| 62 | *ThZF1([Xu et al., 2006](#_ENREF_107))* | ABI74621.1 | ThZF1 involved in drought and salt stress; ThZF1 may have similar roles as *Arabidopsis* AZF2 in plant development as well as regulation of downstream gene. | *Thellungiella halophila* | *ZmZFP105*  *ZmZFP186*  *ZmZFP026*  *ZmZFP012*  *ZmZFP011* |
| 63 | *REF6([Lu et al., 2011](#_ENREF_65); [Cui et al., 2016](#_ENREF_12); [Ko et al., 2010](#_ENREF_49); [Yu et al., 2008](#_ENREF_115); [Li et al., 2016a](#_ENREF_57); [Noh et al., 2004](#_ENREF_77); [Hou et al., 2014](#_ENREF_30))* | AT3G48430 | Histone demethylase that demethylates 'Lys-27' (H3K27me) of histone H3. Demethylates both tri- (H3K27me3) and di-methylated (H3K27me2) H3K27me. Demethylates also H3K4me3/2 and H3K36me3/2 in an in vitro assay. Involved in the transcriptional regulation of hundreds of genes regulating developmental patterning and responses to various stimuli. Binds DNA via its four zinc fingers in a sequence-specific manner, 5'-CTCTGYTY-3', to promote the demethylation of H3K27me3 and the regulation of organ boundary formation. Involved in the regulation of flowering time by repressing FLOWERING LOCUS C (FLC) expression. Interacts with the NF-Y complexe to regulate SOC1. Mediates the recruitment of BRM to its target loci. | *Arabidopsis thaliana* | *ZmZFP076*  *ZmZFP193*  *ZmZFP200*  *ZmZFP001*  *ZmZFP192* |
| 64 | *EMBRYONIC FLOWER 2/EMF2([Yoshida et al., 2001](#_ENREF_113); [Kim et al., 2010](#_ENREF_48); [Calonje et al., 2008](#_ENREF_5))* | AT5G51230 | EMF2 plays a major role in maintain vegetative development and repress flower development; Epigenetic regulation of gene programs by EMF1 and EMF2 in *Arabidopsis*; The plant-specific protein EMBRYONIC FLOWER1 (EMF1) functions in maintaining the repression of the flower homeotic gene AGAMOUS (AG) during vegetative development in *Arabidopsis* thaliana by acting in concert with the EMF2 complex, a putative equivalent of Drosophila melanogaster PRC2. | *Arabidopsis thaliana* | *ZmZFP241.1* |
| 65 | *BoEMF2*(*BoEMF2.1, BoEMF2.2*)*([Liu et al., 2012](#_ENREF_61))* | AFI23583.1, AFI23584.1 | *BoEMF2* regulates genes involved in diverse developmental and stress programs similar to *AtEMF2* in *Arabidopsis*. However, *BoEMF2* differs from *AtEMF2* in the regulation of flower organ identity, cell proliferation and elongation, and death-related genes, which may explain the distinct phenotypes. | Brassica oleracea var. italica | ZmZFP241 |
| 66 | *OsEMF2([Li et al., 2009](#_ENREF_58); [Luo et al., 2009](#_ENREF_67))* | AAQ84239 | The tissue-specific expression pattern of OsEMF2 reveals that it is abundant in shoot apical meristem and inflorescence meristem, while its expression level is much lower in leaf, root, immature seed and callus. | *Oryza sativa* | *ZmZFP241* |
| 67 | *TAC1([Ren et al., 2004](#_ENREF_84); [Ren et al., 2007](#_ENREF_85))* | AT3G09290 | Activation factor which mediates telomerase activity and potentiates responses to auxin through the regulation of BT2. Binds in vitro to the DNA sequence 5'-GACAGTGTTAC-3' of the BT2 promoter. | *Arabidopsis thaliana* | *ZmZFP135* |

References

An, L., Zhou, Z., Su, S., Yan, A., and Gan, Y. (2012a). GLABROUS INFLORESCENCE STEMS (GIS) is required for trichome branching through gibberellic acid signaling in *Arabidopsis*. *Plant Cell Physiol.* 53, 457-69.

An, L., Zhou, Z., Sun, L., Yan, A., Xi, W., Yu, N., et al. (2012b). A zinc finger protein gene *ZFP5* integrates phytohormone signaling to control root hair development in *Arabidopsis*. *The Plant Journal.* 72, 474-490.

Baek, D., Cha, J.-Y., Kang, S., Park, B., Lee, H.-J., Hong, H., et al. (2015). The *Arabidopsis* a zinc finger domain protein *ARS1* is essential for seed germination and ROS homeostasis in response to ABA and oxidative stress. *Frontiers in Plant Science.* 6, 963.

Borg, M., Rutley, N., Kagale, S., Hamamura, Y., Gherghinoiu, M., Kumar, S., et al. (2014). An EAR-dependent regulatory module promotes male germ cell division and sperm fertility in *Arabidopsis*. *The Plant Cell.* 26, 2098-2113.

Calonje, M., Sanchez, R., Chen, L., and Sung, Z. R. (2008). EMBRYONIC FLOWER1 participates in polycomb group-mediated AG gene silencing in *Arabidopsis*. *Plant Cell.* 20, 277-91.

Chang, J., Yu, T., Yang, Q., Li, C., Xiong, C., Gao, S., et al. (2018). *Hair*, encoding a single C2H2 zinc-finger protein, regulates multicellular trichome formation in tomato. *The Plant Journal.* 96, 90-102.

Chen, J., Yang, L., Yan, X., Liu, Y., Wang, R., Fan, T., et al. (2016). Zinc-finger transcription factor ZAT6 positively regulates Cadmium tolerance through the glutathione-dependent pathway in *Arabidopsis*. *Plant Physiology.* 171, 707-19.

Choi, J., Hyun, Y., Kang, M. J., In Yun, H., Yun, J. Y., Lister, C., et al. (2009). Resetting and regulation of Flowering Locus C expression during *Arabidopsis* reproductive development. *Plant Journal.* 57, 918-31.

Choi, K., Kim, J., Hwang, H. J., Kim, S., Park, C., Kim, S. Y., et al. (2011). The FRIGIDA complex activates transcription of FLC, a strong flowering repressor in *Arabidopsis*, by recruiting chromatin modification factors. *Plant Cell.* 23, 289-303.

Chung, K. S., Lee, J. H., Lee, J. S., and Ahn, J. H. (2013). Fruit indehiscence caused by enhanced expression of *NO TRANSMITTING TRACT* in *Arabidopsis thaliana*. *Molecules and cells.* 35, 519-525.

Cui, D., Zhao, J., Jing, Y., Fan, M., Liu, J., Wang, Z., et al. (2013). The *Arabidopsis* IDD14, IDD15, and IDD16 cooperatively regulate lateral organ morphogenesis and gravitropism by promoting auxin biosynthesis and transport. *PLoS Genet.* 9, e1003759.

Cui, X., Lu, F., Qiu, Q., Zhou, B., Gu, L., Zhang, S., et al. (2016). REF6 recognizes a specific DNA sequence to demethylate H3K27me3 and regulate organ boundary formation in *Arabidopsis*. *Nature Genetics.* 48, 694-699.

Devaiah, B. N., Nagarajan, V. K., and Raghothama, K. G. (2007). Phosphate homeostasis and root development in *Arabidopsis* are synchronized by the zinc finger transcription factor ZAT6. *Plant Physiology.* 145, 147-59.

Dinkins, R., Pflipsen, C., Thompson, A., and Collins, G. B. (2002). Ectopic expression of an *Arabidopsis* single zinc finger gene in tobacco results in dwarf plants. *Plant Cell Physiol.* 43, 743-50.

Dinkins, R. D., Pflipsen, C., and Collins, G. B. (2003). Expression and deletion analysis of an *Arabidopsis* SUPERMAN-like zinc finger gene. *Plant Science.* 165, 33-41.

Dinneny, J. R., Weigel, D., and Yanofsky, M. F. (2005). A genetic framework for fruit patterning in *Arabidopsis thaliana*. *Development.* 132, 4687-96.

Dinneny, J. R., Weigel, D., and Yanofsky, M. F. (2006). *NUBBIN* and *JAGGED* define stamen and carpel shape in *Arabidopsis*. *Development.* 133, 1645-1655.

Dinneny, J. R., Yadegari, R., Fischer, R. L., Yanofsky, M. F., and Weigel, D. (2004). The role of *JAGGED* in shaping lateral organs. *Development.* 131, 1101-1110.

Feurtado, J. A., Huang, D., Wicki-Stordeur, L., Hemstock, L. E., Potentier, M. S., Tsang, E. W. T., et al. (2011). The *Arabidopsis* C2H2 zinc finger INDETERMINATE DOMAIN1/ENHYDROUS promotes the transition to germination by regulating light and hormonal signaling during seed maturation. *The Plant Cell.* 23, 1772-1794.

Fukazawa, J., Teramura, H., Murakoshi, S., Nasuno, K., Nishida, N., Ito, T., et al. (2014). DELLAs function as coactivators of *G*AI-ASSOCIATED FACTOR1 in regulation of gibberellin homeostasis and signaling in *Arabidopsis*. *The Plant Cell.* 26, 2920-2938.

Gan, Y., Kumimoto, R., Liu, C., Ratcliffe, O., Yu, H., and Broun, P. (2006). GLABROUS INFLORESCENCE STEMS modulates the regulation by gibberellins of epidermal differentiation and shoot maturation in *Arabidopsis*. *Plant Cell.* 18, 1383-95.

Gan, Y., Liu, C., Yu, H., and Broun, P. (2007). Integration of cytokinin and gibberellin signalling by *Arabidopsis* transcription factors *GIS*, *ZFP8* and *GIS2* in the regulation of epidermal cell fate. *Development.* 134, 2073-2081.

Gao, J., Yan, S., Yu, H., Zhan, M., Guan, K., Wang, Y., et al. (2019). Sweet sorghum (*Sorghum bicolor* L.) *SbSTOP1* activates the transcription of a β-1,3-glucanase gene to reduce callose deposition under Al toxicity: A novel pathway for Al tolerance in plants. *Bioscience, Biotechnology, and Biochemistry.* 83, 446-455.

Gechev, T. S., Minkov, I. N., and Hille, J. (2005). Hydrogen peroxide-induced cell death in *Arabidopsis*: transcriptional and mutant analysis reveals a role of an oxoglutarate-dependent dioxygenase gene in the cell death process. *IUBMB life.* 57, 181-8.

Grewal, S., Caro, E., Stroud, H., Greenberg, M. V. C., Bernatavichute, Y. V., Feng, S., et al. (2012). The SET-domain protein SUVR5 mediates H3K9me2 deposition and silencing at stimulus response genes in a DNA methylation–independent manner. *PLoS Genetics.* 8, e1002995.

Guitton, A. E., Page, D. R., Chambrier, P., Lionnet, C., Faure, J. E., Grossniklaus, U., et al. (2004). Identification of new members of Fertilisation Independent Seed Polycomb Group pathway involved in the control of seed development in *Arabidopsis thaliana*. *Development.* 131, 2971-81.

Han, G., Yuan, F., Guo, J., Zhang, Y., Sui, N., and Wang, B. (2019). *AtSIZ1* improves salt tolerance by maintaining ionic homeostasis and osmotic balance in *Arabidopsis*. *Plant Science.* 285, 55-67.

Han, Y.-Y., Zhou, H.-Y., Xu, L.-A., Liu, X.-Y., Fan, S.-X., and Cao, J.-S. (2018). The zinc-finger transcription factor *BcMF20* and its orthologs in Cruciferae which are required for pollen development. *Biochemical and Biophysical Research Communications.* 503, 998-1003.

Han, Y., Zhang, A., Huang, L., Yu, X., Yang, K., Fan, S., et al. (2011). *BcMF20*, a putative pollen-specific transcription factor from *Brassica campestris* ssp. *chinensis*. *Molecular Biology Reports.* 38, 5321-5325.

Hou, X., Zhou, J., Liu, C., Liu, L., Shen, L., and Yu, H. (2014). Nuclear factor Y-mediated H3K27me3 demethylation of the SOC1 locus orchestrates flowering responses of *Arabidopsis*. *Nat Commun.* 5, 4601.

Huang, S., Gao, J., You, J., Liang, Y., Guan, K., Yan, S., et al. (2018). Identification of STOP1-Like proteins associated with Aluminum tolerance in sweet sorghum (*Sorghum bicolor* L.). *Frontiers in Plant Science.* 9, 258.

Isernia, C., Bucci, E., Leone, M., Zaccaro, L., Lello, P. D., Digilio, G., et al. (2003). NMR structure of the single QALGGH zinc finger domain from the *Arabidopsis thaliana* SUPERMAN protein. *ChemBioChem.* 4, 171-180.

Ito, T., Sakai, H., and Meyerowitz, E. M. (2003). Whorl-specific expression of the *SUPERMAN* gene of *Arabidopsis* is mediated by cis elements in the transcribed region. *Current Biology.* 13, 1524-1530.

Iuchi, S., Koyama, H., Iuchi, A., Kobayashi, Y., Kitabayashi, S., Ikka, T., et al. (2007). Zinc finger protein *STOP1* is critical for proton tolerance in *Arabidopsis* and coregulates a key gene in aluminum tolerance. *Proceedings of the National Academy of Sciences.* 104, 9900-9905.

Jiang, C.-J., Aono, M., Tamaoki, M., Maeda, S., Sugano, S., Mori, M., et al. (2007). *SAZ*, a new SUPERMAN-like protein, negatively regulates a subset of ABA-responsive genes in *Arabidopsis*. *Molecular Genetics and Genomics.* 279, 183-192.

Jin, J. B., Jin, Y. H., Lee, J., Miura, K., Yoo, C. Y., Kim, W. Y., et al. (2008). The SUMO E3 ligase, AtSIZ1, regulates flowering by controlling a salicylic acid-mediated floral promotion pathway and through affects on FLC chromatin structure. *Plant Journal.* 53, 530-40.

Joseph, M. P., Papdi, C., Kozma-Bognár, L., Nagy, I., López-Carbonell, M., Rigó, G., et al. (2014). The *Arabidopsis* ZINC FINGER PROTEIN3 interferes with abscisic acid and light signaling in seed germination and plant development. *Plant Physiology.* 165, 1203-1220.

Jullien, P. E., Kinoshita, T., Ohad, N., and Berger, F. (2006). Maintenance of DNA methylation during the *Arabidopsis* life cycle is essential for parental imprinting. *Plant Cell.* 18, 1360-72.

Kazama, Y., Fujiwara, M. T., Koizumi, A., Nishihara, K., Nishiyama, R., Kifune, E., et al. (2009). A SUPERMAN-like gene is exclusively expressed in female flowers of the dioecious plant *Silene latifolia*. *Plant and Cell Physiology.* 50, 1127-1141.

Kim, J., Jeong, J., Park, H., Yoo, J., Koo, Y., Yoon, H., et al. (2001a). Cold accumulation of SCOF-1 transcripts is associated with transcriptional activation and mRNA stability. *Molecules and Cells.* 12, 204-208.

Kim, J., Lee, S., Cheong, Y., Yoo, C., Lee, S., Chun, H., et al. (2001b). A novel cold-inducible zinc finger protein from soybean, SCOF-1, enhances cold tolerance in transgenic plants. *The Plant Journal.* 25, 247–259.

Kim, J. Y., Jang, I. C., and Seo, H. S. (2016a). COP1 controls abiotic stress responses by modulating *AtSIZ1* function through its E3 ubiquitin ligase activity. *Front Plant Sci.* 7, 1182.

Kim, J. Y., Ryu, J. Y., Baek, K., and Park, C. M. (2015a). High temperature attenuates the gravitropism of inflorescence stems by inducing *SHOOT GRAVITROPISM 5* alternative splicing in *Arabidopsis*. *New Phytologist.* 209, 265-279.

Kim, S., Choi, K., Park, C., Hwang, H.-J., and Lee, I. (2006). *SUPPRESSOR OF FRIGIDA4*, Encoding a C2H2-type zinc finger protein, represses llowering by transcriptional activation of *Arabidopsis* *FLOWERING LOCUS C*. *The Plant Cell.* 18, 2985-2998.

Kim, S. I., Kwak, J. S., Song, J. T., and Seo, H. S. (2016b). The E3 SUMO ligase *AtSIZ1* functions in seed germination in *Arabidopsis*. *Physiologia plantarum.* 158, 256-271.

Kim, S. I., Park, B. S., Kim, D. Y., Yeu, S. Y., Song, S. I., Song, J. T., et al. (2015b). E3 SUMO ligase AtSIZ1 positively regulates SLY1-mediated GA signalling and plant development. *Biochem J.* 469, 299-314.

Kim, S. Y., and Michaels, S. D. (2006). SUPPRESSOR OF FRI 4 encodes a nuclear-localized protein that is required for delayed flowering in winter-annual *Arabidopsis*. *Development.* 133, 4699-707.

Kim, S. Y., Zhu, T., and Sung, Z. R. (2010). Epigenetic regulation of gene programs by EMF1 and EMF2 in *Arabidopsis*. *Plant Physiology.* 152, 516-28.

Ko, J. H., Mitina, I., Tamada, Y., Hyun, Y., Choi, Y., Amasino, R. M., et al. (2010). Growth habit determination by the balance of histone methylation activities in *Arabidopsis*. *EMBO J.* 29, 3208-15.

Krichevsky, A., Gutgarts, H., Kozlovsky, S. V., Tzfira, T., Sutton, A., Sternglanz, R., et al. (2007). C2H2 zinc finger-SET histone methyltransferase is a plant-specific chromatin modifier. *Developmental biology.* 303, 259-269.

Kundu, A., Das, S., Basu, S., Kobayashi, Y., Koyama, H., Ganesan, M., et al. (2018). *GhSTOP1*, a C2H2 type zinc finger transcription factor is essential for aluminum and proton stress tolerance and lateral root initiation in cotton. *Plant Biology.* 21, 35-44.

Kwak, J. S., Kim, S. I., Park, S. W., Song, J. T., and Seo, H. S. (2019). E3 SUMO ligase AtSIZ1 regulates the cruciferin content of *Arabidopsis* seeds. *Biochem Biophys Res Commun.* 519, 761-766.

Lawrence, S. D., and Novak, N. G. (2018a). Over-expression of *StZFP2* in *Solanum tuberosum* L. var. Kennebec (potato) inhibits growth of Tobacco Hornworm larvae (THW, *Manduca sexta* L.). *Plant signaling & behavior.* 13, e1489668.

Lawrence, S. D., and Novak, N. G. (2018b). The remarkable plethora of infestation-responsive Q-type C2H2 transcription factors in potato. *BMC Res Notes.* 11, 398.

Lawrence, S. D., Novak, N. G., Jones, R. W., Farrar, R. R., and Blackburn, M. B. (2014). Herbivory responsive C2H2 zinc finger transcription factor protein StZFP2 from potato. *Plant Physiology and Biochemistry.* 80, 226-233.

Lee, Shin A., Jang, S., Yoon, Eun K., Heo, J.-O., Chang, Kwang S., Choi, Ji W., et al. (2016). Interplay between ABA and GA modulates the timing of asymmetric cell divisions in the *Arabidopsis* root ground tissue. *Molecular Plant.* 9, 870-884.

Li, C., Gu, L., Gao, L., Chen, C., Wei, C. Q., Qiu, Q., et al. (2016a). Concerted genomic targeting of H3K27 demethylase REF6 and chromatin-remodeling ATPase BRM in *Arabidopsis*. *Nature Genetics.* 48, 687-93.

Li, K., Yang, J., Liu, J., Du, X., Wei, C., Su, W., et al. (2009). Cloning, characterization and tissue-specific expression of a cdna encoding a novel *Embryonic Flower 2* Gene (*OsEMF2*) in *Oryza Sativa*. *DNA Sequence.* 17, 74-78.

Li, T., Wu, X.-Y., Li, H., Song, J.-H., and Liu, J.-Y. (2016b). A dual-function transcription factor, *AtYY1*, is a novel negative regulator of the *Arabidopsis* ABA response network. *Molecular Plant.* 9, 650-661.

Li, Y., Chu, Z., Luo, J., Zhou, Y., Cai, Y., Lu, Y., et al. (2018). The C2H2 zinc-finger protein SlZF3 regulates AsA synthesis and salt tolerance by interacting with CSN5B. *Plant Biotechnology Journal.* 16, 1201-1213.

Liu, M.-S., Chen, L.-F. O., Lin, C.-H., Lai, Y.-M., Huang, J.-Y., and Sung, Z. R. (2012). Molecular and functional characterization of broccoli *EMBRYONIC FLOWER 2* genes. *Plant and Cell Physiology.* 53, 1217-1231.

Liu, X.-M., An, J., Han, H. J., Kim, S. H., Lim, C. O., Yun, D.-J., et al. (2014). *ZAT11*, a zinc finger transcription factor, is a negative regulator of nickel ion tolerance in *Arabidopsis*. *Plant Cell Reports.* 33, 2015-2021.

Liu, X.-M., Nguyen, X. C., Kim, K. E., Han, H. J., Yoo, J., Lee, K., et al. (2013). Phosphorylation of the zinc finger transcriptional regulator ZAT6 by MPK6 regulates *Arabidopsis* seed germination under salt and osmotic stress. *Biochemical and Biophysical Research Communications.* 430, 1054-1059.

Liu, Y.-T., Shi, Q.-H., Cao, H.-J., Ma, Q.-B., Nian, H., and Zhang, X.-X. (2020). Heterologous expression of a *Glycine soja* C2H2 zinc finger gene improves Aluminum tolerance in *Arabidopsis*. *International Journal of Molecular Sciences.* 21, 2754.

Lu, F., Cui, X., Zhang, S., Jenuwein, T., and Cao, X. (2011). *Arabidopsis* REF6 is a histone H3 lysine 27 demethylase. *Nature Genetics.* 43, 715-9.

Luo, M., Bilodeau, P., Dennis, E. S., Peacock, W. J., and Chaudhury, A. (2000). Expression and parent-of-origin effects for FIS2, MEA, and FIE in the endosperm and embryo of developing *Arabidopsis* seeds. *Proc Natl Acad Sci U S A.* 97, 10637-42.

Luo, M., Platten, D., Chaudhury, A., Peacock, W., and Dennis, E. (2009). Expression, imprinting, and evolution of rice homologs of the polycomb group genes. *Molecular Plant.* 2, 711-723.

Luo, X., Bai, X., Zhu, D., Li, Y., Ji, W., Cai, H., et al. (2012a). GsZFP1, a new Cys2/His2-type zinc-finger protein, is a positive regulator of plant tolerance to cold and drought stress. *Planta.* 235, 1141-55.

Luo, X., Cui, N., Zhu, Y., Cao, L., Zhai, H., Cai, H., et al. (2012b). Over-expression of *GsZFP1*, an ABA-responsive C2H2-type zinc finger protein lacking a QALGGH motif, reduces ABA sensitivity and decreases stomata size. *Journal of plant physiology.* 169, 1192-1202.

Lyu, T., Hu, Z., Liu, W., and Cao, J. (2019). *Arabidopsis* Cys2/His2 zinc-finger protein *MAZ1* is essential for intine formation and exine pattern. *Biochemical and Biophysical Research Communications.* 518, 299-305.

Marsch-Martínez, N., Zúñiga-Mayo, V. M., Herrera-Ubaldo, H., Ouwerkerk, P. B. F., Pablo-Villa, J., Lozano-Sotomayor, P., et al. (2014). The *NTT* transcription factor promotes replum development in *Arabidopsis* fruits. *The Plant Journal.* 80, 69-81.

Mittler, R., Kim, Y., Song, L., Coutu, J., Coutu, A., Ciftci-Yilmaz, S., et al. (2006). Gain- and loss-of-function mutations in *Zat10* enhance the tolerance of plants to abiotic stress. *FEBS letters.* 580, 6537-6542.

Miura, K., Rus, A., Sharkhuu, A., Yokoi, S., Karthikeyan, A. S., Raghothama, K. G., et al. (2005). The *Arabidopsis* SUMO E3 ligase SIZ1 controls phosphate deficiency responses. *Proc Natl Acad Sci U S A.* 102, 7760-5.

Morita, M. T., Sakaguchi, K., Kiyose, S.-i., Taira, K., Kato, T., Nakamura, M., et al. (2006). A C2H2-type zinc finger protein, SGR5, is involved in early events of gravitropism in *Arabidopsis* inflorescence stems. *The Plant Journal.* 47, 619-628.

Nakagawa, H., Ferrario, S., Angenent, G. C., Kobayashi, A., and Takatsuji, H. (2004). The Petunia ortholog of *Arabidopsis* *SUPERMAN* plays a distinct role in floral organ morphogenesis. *The Plant Cell.* 16, 920-932.

Nguyen, X. C., Kim, S. H., Lee, K., Kim, K. E., Liu, X.-M., Han, H. J., et al. (2011). Identification of a C2H2-type zinc finger transcription factor (ZAT10) from *Arabidopsis* as a substrate of MAP kinase. *Plant Cell Reports.* 31, 737-745.

Noh, B., Lee, S. H., Kim, H. J., Yi, G., Shin, E. A., Lee, M., et al. (2004). Divergent roles of a pair of homologous jumonji/zinc-finger-class transcription factor proteins in the regulation of *Arabidopsis* flowering time. *Plant Cell.* 16, 2601-13.

Ohno, C. K., Reddy, G. V., Heisler, M. G. B., and Meyerowitz, E. M. (2004). The *Arabidopsis* *JAGGED* gene encodes a zinc finger protein that promotes leaf tissue development. *Development.* 131, 1111-1122.

Park, B. S., Song, J. T., and Seo, H. S. (2011). *Arabidopsis* nitrate reductase activity is stimulated by the E3 SUMO ligase AtSIZ1. *Nat Commun.* 2, 400.

Payne, T., Johnson, S. D., and Koltunow, A. M. (2004). *KNUCKLES* (*KNU*) encodes a C2H2 zinc-finger protein that regulates development of basal pattern elements of the *Arabidopsis* gynoecium. *Development.* 131, 3737-3749.

Prigge, M. J., and Wagner, D. R. (2001). The *Arabidopsis* *SERRATE* gene encodes a zinc-finger protein required for normal shoot development. *The Plant Cell.* 13, 1263–1279.

Prunet, N., Yang, W., Das, P., Meyerowitz, E., and Jack, T. (2017). *SUPERMAN* prevents class B gene expression and promotes stem cell termination in the fourth whorl of *Arabidopsis thaliana* flowers. *Proc Natl Acad Sci USA.* 114, 7166-7171.

Qiu, Y., Liu, S. L., and Adams, K. L. (2017). Concerted divergence after gene duplication in polycomb repressive complexes. *Plant Physiology.* 174, 1192-1204.

Ren, S., Johnston, J. S., Shippen, D. E., and McKnight, T. D. (2004). *TELOMERASE ACTIVATOR1* induces telomerase activity and potentiates responses to auxin in *Arabidopsis*. *The Plant Cell.* 16, 2910-2922.

Ren, S., Mandadi, K. K., Boedeker, A. L., Rathore, K. S., and McKnight, T. D. (2007). Regulation of telomerase in *Arabidopsis* by BT2, an apparent target of *TELOMERASE ACTIVATOR1*. *The Plant Cell.* 19, 23-31.

Resentini, F., Cyprys, P., Steffen, J. G., Alter, S., Morandini, P., Mizzotti, C., et al. (2017). *SUPPRESSOR OF FRIGIDA* (*SUF4*) supports gamete fusion via regulating *Arabidopsis* *EC1* gene expression. *Plant Physiology.* 173, 155-166.

Royo, J., Gómez, E., Barrero, C., Muñiz, L. M., Sanz, Y., and Hueros, G. (2009). Transcriptional activation of the maize endosperm transfer cell-specific gene *BETL1* by *ZmMRP-1* is enhanced by two C2H2 zinc finger-containing proteins. *Planta.* 230, 807-818.

Sakai, H., Medrano, L., and Meyerowitz, E. (1995). Role of *SUPERMAN* in maintaining *Arabidopsis* floral whorl boundaries. *Nature Biotechnology.* 378, 199-203.

Sawaki, Y., Iuchi, S., Kobayashi, Y., Kobayashi, Y., Ikka, T., Sakurai, N., et al. (2009). *STOP1* regulates multiple genes that protect *Arabidopsis* from proton and aluminum toxicities. *Plant Physiology.* 150, 281-294.

Shi, H., Liu, G., Wei, Y., and Chan, Z. (2018a). The zinc-finger transcription factor ZAT6 is essential for hydrogen peroxide induction of anthocyanin synthesis in *Arabidopsis*. *Plant Mol Biol.* 97, 165-176.

Shi, X., Gu, Y., Dai, T., Wu, Y., Wu, P., Xu, Y., et al. (2018b). Regulation of trichome development in tobacco by *JcZFP8* , a C2H2 zinc finger protein gene from *Jatropha curcas* L. *Gene.* 658, 47-53.

Song, S.-K., Jang, H.-U., Kim, Y. H., Lee, B. H., and Lee, M. M. (2020). Overexpression of three related root-cap outermost-cell-specific C2H2-type zinc-finger protein genes suppresses the growth of *Arabidopsis* in an EAR-motif-dependent manner. *BMB Reports.* 53, 160-165.

Sun, L.-l., Zhou, Z.-j., An, L.-j., An, Y., Zhao, Y.-q., Meng, X.-f., et al. (2013). *GLABROUS INFLORESCENCE STEMS* regulates trichome branching by genetically interacting with SIM in *Arabidopsis*. *Journal of Zhejiang University SCIENCE B.* 14, 563-569.

Sun, L., Zhang, A., Zhou, Z., Zhao, Y., Yan, A., Bao, S., et al. (2015). GLABROUS INFLORESCENCE STEMS3 (GIS3) regulates trichome initiation and development in *Arabidopsis*. *The New phytologist.* 206, 220-230.

Sun, Y., Yang, Y., Yuan, Z., Müller, J. L., Yu, C., Xu, Y., et al. (2010). Overexpression of the *Arabidopsis* gene *UPRIGHT ROSETTE* reveals a homeostatic control for indole-3-acetic acid. *Plant Physiology.* 153, 1311-1320.

Sun, Z., Liu, R., Guo, B., Huang, K., Wang, L., Han, Y., et al. (2019). Ectopic expression of *GmZAT4*, a putative C2H2-type zinc finger protein, enhances PEG and NaCl stress tolerances in *Arabidopsis thaliana*. *3 Biotech.* 9, 166.

Takeda, S., Matsumoto, N., and Okada, K. (2004). RABBIT EARS, encoding a SUPERMAN-like zinc finger protein, regulates petal development in *Arabidopsis thaliana*. *Development.* 131, 425-34.

Tang, L., Cai, H., Ji, W., Luo, X., Wang, Z., Wu, J., et al. (2013). Overexpression of *GsZFP1* enhances salt and drought tolerance in transgenic alfalfa (*Medicago sativa* L.). *Plant Physiology and Biochemistry.* 71, 22-30.

Tanimoto, M., Tremblay, R., and Colasanti, J. (2008). Altered gravitropic response, amyloplast sedimentation and circumnutation in the *Arabidopsis* shoot gravitropism 5 mutant are associated with reduced starch levels. *Plant Mol Biol.* 67, 57-69.

Teng, K., Tan, P., Guo, W., Yue, Y., Fan, X., and Wu, J. (2018). Heterologous expression of a novel *Zoysia japonica* C2H2 zinc finger gene, *ZjZFN1*, improved salt tolerance in *Arabidopsis*. *Frontiers in Plant Science.* 9, 1159.

Tian, Z. D., Zhang, Y., Liu, J., and Xie, C. H. (2010). Novel potato C2H2-type zinc finger protein gene, *StZFP1*, which responds to biotic and abiotic stress, plays a role in salt tolerance. *Plant Biology.* 12, 689-697.

Tsutsui, T., Yamaji, N., and Ma, J. F. (2011). Identification of a cis-acting element of ART1, a C2H2-type zinc-finger transcription factor for aluminum tolerance in rice. *Plant Physiology.* 156, 925-931.

Weingartner, M., Subert, C., and Sauer, N. (2011). LATE, a C2H2 zinc-finger protein that acts as floral repressor. *The Plant Journal.* 68, 681-692.

Wu, K., Zhao, J., Liu, M., Jiang, L., Ding, L., Yan, S. S., et al. (2014). Cucumber *SUPERMAN* has conserved function in stamen and fruit development and a distinct role in floral patterning. *PLoS ONE.* 9, e86192.

Xiao, H., Tang, J., Li, Y., Wang, W., Li, X., Jin, L., et al. (2009). *STAMENLESS 1*, encoding a single C2H2 zinc finger protein, regulates floral organ identity in rice. *The Plant Journal.* 59, 789-801.

Xu, B., Li, Z., Zhu, Y., Wang, H., Ma, H., Dong, A., et al. (2008). *Arabidopsis* genes AS1, AS2, and JAG negatively regulate boundary-specifying genes to promote sepal and petal development. *Plant Physiology.* 146, 566-75.

Xu, S., Wang, X., and Chen, J. (2006). Zinc finger protein 1 (*ThZF1*) from salt cress (*Thellungiella halophila*) is a Cys-2/His-2-type transcription factor involved in drought and salt stress. *Plant Cell Reports.* 26, 497-506.

Xu, Y., Prunet, N., Gan, E. S., Wang, Y., Stewart, D., Wellmer, F., et al. (2018). *SUPERMAN* regulates floral whorl boundaries through control of auxin biosynthesis. *The EMBO journal.* 37, e97499.

Yamaji, N., Huang, C. F., Nagao, S., Yano, M., Sato, Y., Nagamura, Y., et al. (2009). A zinc finger transcription factor ART1 regulates multiple genes implicated in Aluminum tolerance in rice. *The Plant Cell.* 21, 3339-3349.

Yamauchi, Y., Fukaki, H., Fujisawa, H., and Tasaka, M. (1997). Mutations in the SGR4, SGR5 and SGR6 loci of *Arabidopsis thaliana* alter the shoot gravitropism. *Plant Cell Physiol.* 38, 530-5.

Yang, L., Liu, Z., Lu, F., Dong, A., and Huang, H. (2006). *SERRATE* is a novel nuclear regulator in primary microRNA processing in *Arabidopsis*. *The Plant Journal.* 47, 841-850.

Yin, M., Wang, Y., Zhang, L., Li, J., Quan, W., Yang, L., et al. (2017). The *Arabidopsis* Cys2/His2 zinc finger transcription factor ZAT18 is a positive regulator of plant tolerance to drought stress. *Journal of Experimental Botany.* 68, 2991-3005.

Yoshida, N., Yanai, Y., Chen, L., Kato, Y., Hiratsuka, J., Miwa, T., et al. (2001). *EMBRYONIC FLOWER2*, a novel polycomb group protein homolog, mediates shoot development and flowering in *Arabidopsis*. *The Plant Cell.* 13, 2471-2481.

Yu, G. H., Jiang, L. L., Ma, X. F., Xu, Z. S., Liu, M. M., Shan, S. G., et al. (2014). A soybean C2H2-type zinc finger gene *GmZF1* enhanced cold tolerance in transgenic *Arabidopsis*. *PLoS ONE.* 9, e109399.

Yu, X., Li, L., Guo, M., Chory, J., and Yin, Y. (2008). Modulation of brassinosteroid-regulated gene expression by Jumonji domain-containing proteins ELF6 and REF6 in *Arabidopsis*. *Proc Natl Acad Sci U S A.* 105, 7618-23.

Yue, S., Zhang, W., Li, F. L., Guo, Y. L., Liu, T. L., and Huang, H. (2000). Identification and genetic mapping of four novel genes that regulate leaf development in *Arabidopsis*. *Cell Res.* 10, 325-35.

Yun, J., Weigel, D., and Lee, I. (2002). Ectopic expression of *SUPERMAN* suppresses development of petals and stamens. *Plant Cell Physiology.* 43, 52-57.

Zhang, D., Tong, J., Xu, Z., Wei, P., Xu, L., Wan, Q., et al. (2016). Soybean C2H2-type zinc finger protein GmZFP3 with conserved QALGGH motif negatively regulates drought responses in transgenic *Arabidopsis*. *Frontiers in Plant Science.* 7, 325.

Zhou, Z., An, L., Sun, L., and Gan, Y. (2012). ZFP5 encodes a functionally equivalent GIS protein to control trichome initiation. *Plant signaling & behavior.* 7, 28-30.

Zhou, Z., An, L., Sun, L., Zhu, S., Xi, W., Broun, P., et al. (2011). *Zinc Finger Protein5* is required for the control of trichome initiation by acting upstream of *Zinc Finger Protein8* in *Arabidopsis*. *Plant Physiology.* 157, 673-682.

Zhou, Z., Sun, L., Zhao, Y., An, L., Yan, A., Meng, X., et al. (2013). *Zinc Finger Protein 6* (*ZFP6*) regulates trichome initiation by integrating gibberellin and cytokinin signaling in *Arabidopsis thaliana*. *New Phytologist.* 198, 699-708.
